# Host-Derived Geranylgeraniol Shields Intraerythrocytic Stages of Malaria Parasites from Fosmidomycin

**DOI:** 10.21203/rs.3.rs-6574048/v1

**Published:** 2025-06-05

**Authors:** Ignasi Verdaguer, Cathy Chen, Maurício Mazzine Filho, Matheus Santos, Gabriela Castro, Lydia Yamaguchi, Sandra de Oliveira, Manoel Peres, Agustín Hernández, Thales Kronenberger, Daniel Bargieri, Claudia Angeli, Dana Hodge, Giuseppe Palmisano, Luis Izquierdo, Luciana Azevedo, Audrey Odom John, Alejandro Katzin, Marcell Crispim

**Affiliations:** University of São Paulo; Children’s Hospital of Philadelphia; University of São Paulo; University of São Paulo; University of São Paulo; Universidade de São Paulo; Institute for Technological Research of São Paulo State; University of São Paulo; Betternostics S.L.; University Hospital of Tübingen; University of Sao Paulo; University of São Paulo; Children’s Hospital of Philadelphia; University of Sao Paulo; ISGlobal; Federal University of Alfenas; Children’s Hospital of Pennsylvania; University of Sao Paulo/ Institue of Biomedical Sciences; Federal University of Alfenas

**Keywords:** Prenols, geranylgeraniol, farnesol, fosmidomycin, geraniol, knockout, Plasmodium falciparum, Plasmodium berghei, fosmidomycin, malaria, farnesol/geranylgeraniol kinase, isoprenoid biosynthesis, protein prenylation

## Abstract

Fosmidomycin was proposed as an antimalarial drug but failed in clinical trials due to recrudescence, a phenomenon whose causes remain poorly understood. The mechanism of action of fosmidomycin is the inhibition of the methylerythritol 4-phosphate (MEP) pathway, essential for producing isoprenoids in *Plasmodium* parasites. The key isoprenoids produced by the MEP pathway are farnesyl and geranylgeranyl pyrophosphates (FPP and GGPP), vital for protein isoprenylation, ubiquinone, and dolichol biosynthesis. *In vitro* studies have demonstrated that prenols, like farnesol (FOH) and geranylgeraniol (GGOH), can temporarily circumvent the MEP pathway, rescuing parasites from fosmidomycin effects. Our group identified a parasitic prenol kinase (PolK), responsible for converting FOH and GGOH into their active pyrophosphate forms. Additionally, GGOH’s human plasma concentration is sufficient to affect MEP inhibitors. This suggests that the parasite’s uptake of host prenols could diminish fosmidomycin effectiveness against malaria. To test this hypothesis, we generated *Pb*PolK knockout *P. berghei* parasites (Δ*Pb*PolK). These transgenic parasites were viable but could not utilize exogenous prenols for protein prenylation and caused a form of murine malaria that responded more effectively to fosmidomycin therapy compared to parasites preserving PbPolK. Consequently, we explored compounds that could inhibit the parasite utilization of exogenous prenols, using biochemical and bioinformatics approaches, as well as in vitro assays in wild type and ΔPfPolK P. falciparum strains. Geraniol inhibits PolK activity and the incorporation of GGOH into P. falciparum. Moreover, geraniol enhanced fosmidomycin antimalarial effect in *P. falciparum in vitro*, even in the presence of GGOH. Δ*Pf*PolK *P. falciparum* strains exhibited profound metabolic dysregulation in carbon metabolism, as assessed by proteomics. Taking all together, findings here presented demonstrate that the prenol salvage pathway is a modulatory mechanism of metabolic homeostasis, facilitates prenol utilization from the host, and contributes to the limited efficacy of fosmidomycin in malaria treatment.

## 1. Introduction

*Plasmodium falciparum* is the causative agent of the most severe form of human malaria. In 2023, the World Health Organization reported an estimated 263 million cases of malaria and 597,000 malaria-related deaths. Resistance to current antimalarial drugs is probably the most significant challenge to controlling malaria (WHO, 2024). Therefore, the identification and development of novel antimalarial therapies is urgently needed.

**Figure 1:**
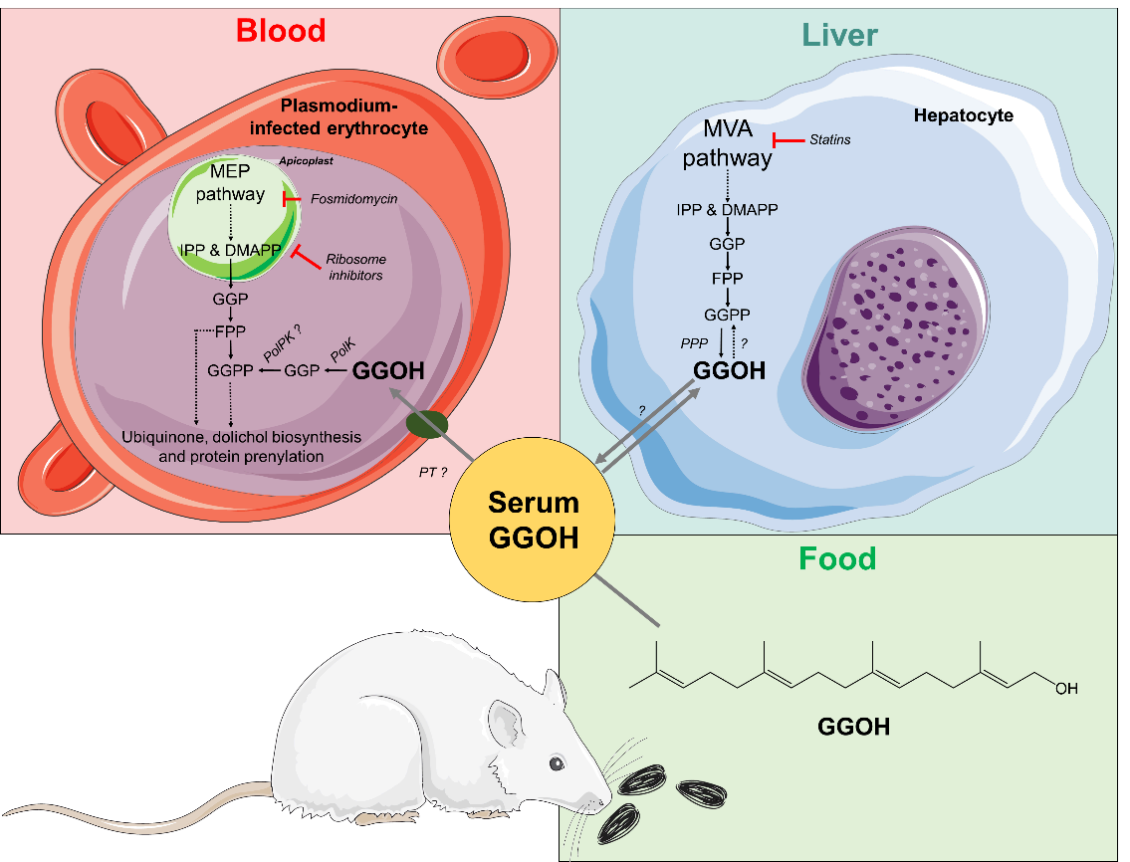
Isoprenoid sources and distribution in malaria parasites, animals and food. The figure illustrates the overall hypothesis of the present article together with already demonstrated sources and distribution of isoprenoids in malaria parasites, animals, and food. The figure includes the biosynthesis of isoprenoids via the MEP pathway and MVA pathway, which leads to the formation of IPP and DMAPP. These molecules are condensed to form GPP, FPP, and GGPP, required for the biosynthesis of ubiquinone, dolichol, and protein prenylation. The figure also shows the mammalian and parasitic prenol salvage pathway and the activity of the mammalian PPP. Additionally, it represents the FOH/GGOH feeding route for animals and malaria parasites (the initial hypothesis of this manuscript). The figure also displays the targets of fosmidomycin, ribosome inhibitors and statins. Finally, the chemical structure of GGOH is represented. Gray arrows indicate GGOH transport between food, blood serum, the malaria parasite, and animal cells; red arrows indicate drug targets; black arrows indicate one (continuous arrow) or more than one enzymatic step (discontinuous arrows); the symbol “?” indicates suspected processes that are not yet well characterized; letters in italics represent enzymes. Abbreviations contained in the figure and a better explanation of its content are in the text. The images were purchased and adapted from Servier Medical Art by Servier (https://smart.servier.com), last accessed in march 2025). Chemical structures were designed using ChemDraw Software.

*Plasmodium* spp. belong to the group of Apicomplexan parasites. Ancestors of this clade of microorganisms underwent an endosymbiosis event with a red alga and thus actual parasites possess a non-photosynthetic plastid called apicoplast and plant-like metabolic pathways (Köhler et al, 1997). The most extensively studied plant-like metabolic pathway in the apicoplast is isoprenoid biosynthesis via the methylerythritol phosphate (MEP) pathway (Fig. 1), a counterpart to the animal mevalonate (MVA) pathway, which has been widely studied for its role in cholesterol biosynthesis and as the target of statins (Seeber & Soldati-Favre, 2010). The MEP pathway starts by the condensation of a pyruvate and a glyceraldehyde 3-phosphate molecule and leads to the production of the 5-carbon isoprene units, isopentenyl pyrophosphate (IPP) and its isomer, dimethylallyl pyrophosphate (DMAPP). IPP and DMAPP are enzymatically condensed in geranyl pyrophosphate (GPP, 10 carbon), farnesyl pyrophosphate (FPP, 15 carbon), geranylgeranyl pyrophosphate (GGPP, 20 carbon), or longer polyprenyl-PP such as octaprenyl-PP or nonaprenyl-PP (Cassera et al, 2004). FPP and GGPP are essential for the farnesylation and geranylgeranylation of proteins and serve as precursors in the biosynthesis of other metabolites, such as ubiquinone or dolichol (Fig 1) (Gowda & Davidson, 1999; de Macedo et al, 2002; Verdaguer et al, 2019; Zimbres et al, 2020; Verdaguer et al, 2021; Fenollar et al, 2022; Okada et al, 2022). The antimalarial fosmidomycin specifically inhibits the MEP pathway by targeting the enzyme 1-deoxy-D-xylulose 5-phosphate reductoisomerase (Lell et al, 2003). Similarly, some bacterial ribosome inhibitors such as azithromycin or clindamycin, can also indirectly inhibit isoprenoid biosynthesis by interfering with apicoplast biogenesis and thus, leading to the fragmentation of the organelle into organoids which cannot maintain active the MEP pathway (Swift et al, 2021; Okada et al., 2022).

Fosmidomycin, either alone or in combination with clindamycin, has failed due to poor antimalarial activity and recrudescence phenomena in clinical trials (Lanaspa et al, 2012; Fernandes et al, 2015; Mombo-Ngoma et al, 2018). Importantly, no mutations related to fosmidomycin resistance had been detected in clinical assays (Guggisberg et al, 2016). Some authors have suggested that this failure is probably due to issues with the pharmacokinetics of fosmidomycin (Na-Bangchang, et al., 2007; Walker, 2018) or intrinsic mechanisms of the parasite (Guggisberg et al, 2016). Considering the last hypothesis, several authors have tried to better understand isoprenoid metabolism in malaria parasites, pursuing the development of effective antimalarial treatments targeting isoprenoid metabolism. One of the most relevant discoveries on this topic is the MEP pathway’s regulatory mechanism (Guggisberg et al., 2014; Park et al., 2015; Frasse & Odom, 2019; Tewari et al., 2021). Researchers maintained *P. falciparum* parasites under fosmidomycin treatment to select drug-resistant strains. Fosmidomycin-resistant parasites exhibited genetic changes in members of the haloacid dehalogenase (HAD) superfamily such as HAD1 and HAD2. The name of this enzyme superfamily comes from its first characterized member, a dehalogenase that acted on haloacid compounds; however, most HAD enzymes actually function as phosphatases, acting on several sugars including glycolysis intermediates. While HAD2-mutants were still viable, these parasites showed reduced growth (Guggisberg et al., 2014). Interestingly, once fosmidomycin was withdrawn, mutant parasites acquired mutations in phosphofructokinase, which simultaneously restored parasitic growth and fosmidomycin sensitivity. Further studies showed that parasites possessing deficiency in glyceraldehyde 3-phosphate dehydrogenase activity possess an increased resistance to fosmidomycin (Jezewski et al., 2022). Taking all together, these data indicate that parasites downregulate the MEP pathway by reducing glycolysis and, consequently, reducing the availability of MEP precursors. However, it remains unclear the biological signal that naturally indicates the fulfillment of isoprenoid requirements and initiates HAD-mediated regulation of the glycolysis/MEP pathway.

Other researchers focused on understanding the cause of death of parasites exposed to apicoplast disruptors. Metabolomics profiling revealed that the lack of ubiquinone and dolichol biosynthesis is not the primary cause of death of parasites exposed to ribosome inhibitors, but the disruption of the digestive vacuole function (Kennedy et al, 2019). This is probably due the lack of prenylation of Ras, Rho, and Rap small GTPases involved in cellular signalling and intracellular trafficking to the digestive vacuole (Howe et al., 2013; Suazo et al, 2016; Verdaguer et al, 2022a). In concordance with the mechanism of action of these drugs, it has been demonstrated that parasites can be grown indefinitely *in vitro* under the presence of fosmidomycin or ribosome inhibitors if high amounts of IPP are supplied (≥100 μM). Similarly, parasites exposed *in vitro* to fosmidomycin or ribosome inhibitors can be rescued for two parasitic cycles by the addition of FPP/farnesol (FOH), GGPP/geranylgeraniol (GGOH) or food extracts rich in this prenols, but not isopentenol, geraniol, octaprenol, nonaprenol or dolichols (Yeh & DeRisi, 2011; Wu et al, 2015; Kennedy et al, 2019; Verdaguer et al, 2022a). Prenols and prenyl-PPs are probably unable to rescue the parasites for more than two cycles because they cannot restore the IPP pool required for the biosynthesis of ubiquinones and glycoconjugates, which are essential in the long term (Verdaguer et al, 2021; Bulloch et al, 2024). In fact, we have already observed an efficient parasitic transport of FOH and GGOH, with a saturable profile of temporal incorporation that adjusts to an exponential decay function. Furthermore, we demonstrated that living *P. falciparum* parasites phosphorylate prenols, condense them into longer prenyl-PPs, and use them for protein prenylation and dolichyl phosphate biosynthesis (Verdaguer et al, 2022a). All this data suggests that the parasite must possess a prenol transporter (PT) and also make evident the existence of a phosphorylative salvage pathway to incorporate prenols into isoprenoid metabolism (Zhang et al., 2011; Yeh & DeRisi, 2011; Howe et al., 2013; Guggisberg et al., 2014; Kennedy et al., 2019; Verdaguer et al., 2022a).

The phosphorylation of prenols has been observed in membrane extracts of animal and plant tissues, as well as in archaea (Verdaguer et al., 2022b). In all cases, there are biochemical indications that this pathway is carried out through the catalytic activity of a prenol kinase (PolK) followed by a prenyl-phosphate kinase (PolPK). Since recently, only a few genes encoding these enzymes were experimentally identified in photosynthetic organisms (Valentin et al., 2006; Fitzpatrick et al., 2011; Vom Dorp et al., 2015). Currently, we identified a gene in malaria parasites that encodes a CTP-dependent prenol kinase (PolK) genetically related to its homologous enzymes in plants and green algae (Crispim et al., 2024). This enzyme phosphorylates FOH and GGOH to farnesyl monophosphate (FP) and geranylgeranyl monophosphate (GGP), respectively (Crispim et al., 2024). Conditional gene expression knockout *P. falciparum* (Δ*Pf*PolK) parasites demonstrated to be viable but more susceptible to fosmidomycin. Furthermore, their sensitivity to MEP inhibitors could not be rescued by the addition of FOH or GGOH, as these substances could no longer being used for protein prenylation (Crispim et al., 2024). Furthermore, it was observed that parasites cultured in the presence of delipidated plasma are more sensitive to fosmidomycin compared to those cultured in whole plasma. However, the type of plasma has no effect on fosmidomycin sensitivity in Δ*Pf*PolK parasites. Through mass spectrometry techniques, GGOH was identified in human blood plasma (Crispim et al., 2024), which may come from dietary sources or be produced by animal prenyl-PP phosphatase (PPP) (Miriyala et al., 2010; Elsabrouty et al., 2021). Considering all these findings, we hypothesized that the prenol phosphorylation pathway could be a regulatory mechanism for the isoprenoid biosynthetic pathway, enable the use of exogenous isoprenoids, and potentially limit the efficacy of fosmidomycin in malaria treatment (Crispim et al., 2024).

To address this issue, this study focused on assessing whether the rodent parasite *P. berghei* FOH/GGOH salvage pathway impacts the *in vivo* efficacy of fosmidomycin. For this, we employed as a tool *Pb*PolK knockout (Δ*Pb*PolK) *P. berghei* parasites, which are unable to use exogenous prenols. Furthermore, we also searched for a PolK inhibitor as a pharmacological strategy to improve fosmidomycin efficacy for malaria treatment. Finally, we explored the biological role of the parasitic prenol salvage pathway.

## 2. RESULTS

### 2.1 Assessing GGPP/GGOH Levels in Plasma and Diet

**Figure 2:**
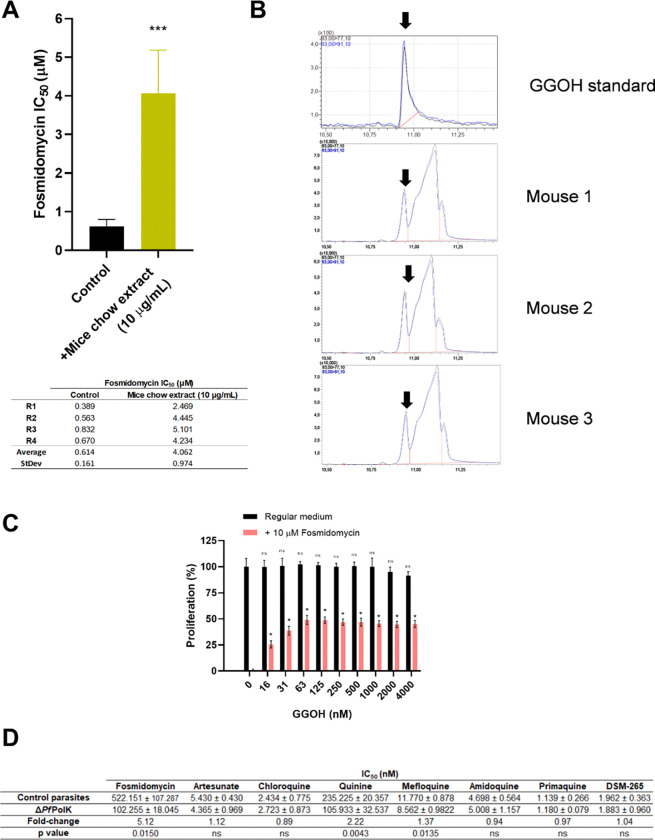
Fosmidomycin sensitivity modulated by mice chow lipids and prenol detection in plasma. (A) Effect of mouse chow extract on fosmidomycin-dependent growth inhibition. *P. falciparum* 3D7 strain was cultured for 72 h in the presence or absence of 10 μg/mL mouse chow extract and increasing concentrations of fosmidomycin. IC_50_ values are shown in the table. Values represent the mean ± SD from three independent assays. (B) GC-MS triple quadrupole MRM analysis showing the GGOH standard and components detected in unsaponifiable lipids from mouse plasma (three individual samples). GGOH is indicated by arrows. (C) Parasite proliferation in the presence of increasing concentrations of GGOH in regular media or media supplemented with 20 μM fosmidomycin. Comparisons were made with the control condition (no GGOH). (D) IC_50_ values of several drugs against control or Δ*Pf*PolK transgenic *P. falciparum* parasites after 72 h. Comparisons were made between *Pf*PolK and Δ*Pf*PolK for each drug (n = 3). Statistical analyses were performed using unpaired Student’s t-test in all comparisons; *p < 0.05, *p < 0.001, ns = not significant.

As commented, the primary hypothesis of this work was that malaria parasites may use prenols from host, coming from dietary sources or animal metabolism. In fact, we previously demonstrated that *P. falciparum* 3D7 parasites can be *in vitro* rescued from fosmidomycin effects by extracts from arugula and sunflower oil (Verdaguer et al., 2022a). Given this information, we sought to determine if the standard mouse chow used in our research centre facilities -composed by whole corn, soybean meal, wheat bran, and vitamins- also contains lipids capable of counteracting the effects of fosmidomycin on malaria parasites. To test this hypothesis, we examined the *in vitro* antiparasitic effects of fosmidomycin on *P. falciparum*, either with or without the presence of 10 μg/μL of unsaponifiable lipid extract from mouse chow (Fig 2A). While the average calculated IC_50_ value for fosmidomycin alone was 0.614 ± 0.161 μM, it was increased 6.62 × fold (to 4.062 ± 0.974 μM) under the presence of unsaponifiable lipid extract from mouse chow. Notably, 10 μg/mL unsaponifiable lipids of mouse chow did not produce any toxic effects on the parasite after 72 h. Upon observing this, we questioned whether FOH or GGOH was present in mouse plasma. The unsaponifiable lipids from three samples of mouse plasma (uninfected with malaria parasites) were analysed using MRM (multiple-reaction monitoring) in triple quadrupole Gas chromatography–mass spectrometry (GC-MS) (Fig 2B). Results revealed for the first time that GGOH is present in mouse plasma, while FOH is either not present or is at concentrations below the detection limit this method. Because the co-elution of GOH with other substances, it was not possible to quantify its concentration in mouse plasma.

We exposed *P. falciparum* parasites to several concentrations of GGOH in regular media or media supplemented with 10 μM fosmidomycin—a concentration close to the *Cmax* observed in clinical trials (Na-Bangchang et al., 2007). The results demonstrated that 10 μM completely killed the parasites, and that GGOH, at 4–0.06 μM, rescued 50% of proliferation. However, GGOH still significantly reduced fosmidomycin efficacy and allowed some parasitic growth, even at concentrations as low as 16 nM (Figure 2C). These findings complement previously discussed studies using whole or delipidated plasma (Crispim et al., 2024), strongly suggesting that blood levels of GGOH are sufficient to reduce fosmidomycin efficacy *in vivo*. Nonetheless, it is important to note that the origin of GGPP/GGOH (whether from endogenous synthesis or dietary sources), as well as the kinetics of its excretion in animals, remains largely unknown. Therefore, blood concentrations of these substances may fluctuate significantly over time.

We sought to further investigate how the deletion of PolK, the enzyme involved in GGOH assimilation might affect the efficacy of various drugs beyond fosmidomycin. For this purpose, we utilized the inducible *Pf*PolK knockout strain of *P. falciparum* (*Crispim et al., 2024*), in which the excision of the *Pf*PolK gene was either not induced (control parasites) or induced (Δ*Pf*PolK). We assessed the IC_50_ values of several widely used antimalarial drugs, as well as some promising experimental compounds, in transgenic *P. falciparum* parasites either maintaining or lacking PolK (Patrick, 2020). Specifically, the tested drugs included artesunate, which generates reactive oxygen species and free radicals, disrupts hemoglobin digestion, and interferes with the ubiquitin-proteasome pathway; aminoquinolines such as quinine, mefloquine, amodiaquine, chloroquine, and primaquine, which interfere with heme metabolism; and DSM-265, an experimental dihydroorotate dehydrogenase inhibitor (Thakare et al., 2019). Most drugs did not show significant changes in efficacy. As expected, fosmidomycin exhibited a significant 5.5-fold reduction in its IC_50_ value in knockout parasites compared to control parasites. Surprisingly, also quinine and, to a lesser extent, mefloquine, showed a significant change, with a 2.2-fold and 1.4-fold reduction in their IC_50_ values, respectively. Regardless, this experiment identified several drugs whose efficacy, at first glance, does not change with PolK deletion. These drugs can, therefore, be used as controls in future studies involving the transgenic *P. berghei* parasites we intend to generate.

### 2.2 Generation of *Pb*PolK knockout parasites unable to use exogenous prenols

**Figure 3:**
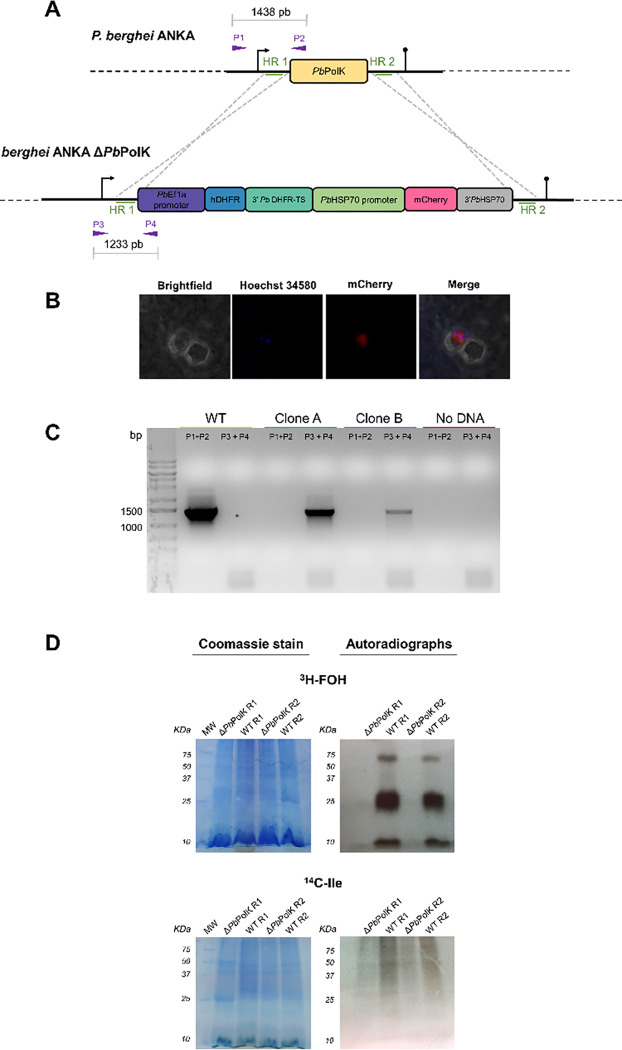
Generation of transgenic *P. berghei* parasites unable to employ exogenous prenols. (A) Illustration of the strategy used for replacing the coding sequence of *Pb*PolK. Two homology regions (HR) were amplified from *P. berghei* gDNA and further cloned in a DcOmCherry plasmid to promote homologous recombination of PolK 5′UTR (962 bp; referred as HR1) and 3′UTR (848 bp; referred as HR2) flanking a cassette for detection/selection of transgenic parasites. Specifically, the cassette contains the pyrimethamine selection marker, the human dihydrofolate reductase (hDHFR), flanked by a promoter (*Pb*Ef1a promoter) and termination sequence (3’ *Pb*DHFR-TS). In order to detect parasites that have integrated the recombinant sequence, the cassette also contains a gene encoding the red fluorescent protein mCherry flanked by *P. berghei* endogenous HSP70 promoter (*Pb*HSP70 promoter) and termination sequence (3’ *Pb*HSP70). The primers (arrowheads) and homology regions (HR; green bars) used for genotyping are shown. The detection of native *Pb*PolK gene was performed by PCR using the primer pair *Pb*PolK-WT-forward (P1) and *Pb*PolK-WT-reverse (P2) which amplified the expected 1438 bp sequence only in parental parasites. The detection of recombinant locus containing mCherry sequence was performed by PCR using the primer pair Int-Δ*Pb*PolK-forward (P3) and Int-Δ*P*bPolK-reverse (P4) which amplified a 1233 bp sequence only in transgenic parasites. It was employed the GeneRuler 1 kb Plus DNA Ladder (#MK-130, Cellco). (B) The image shows the fluorescence microscopy of blood. Infected red blood cells are red-fluorescent (mCherry) and nucleus are blue (DAPI). (C) PCR analysis of the *Pb*PolK locus in parental or mutant clones. P1/P2 pair of primers is specific to the native locus, and P1/P3 pair is specific to integration of the targeting sequence, as better explained in text. (D) Figure shows SDS-PAGE analysis of [^14^C] isoleucine or [^3^H] FOH-labelled proteins of control parasites or Δ*Pb*PolK *P. berghei* parasites cultured *ex vivo*. Figure shows both the Coomassie stained gel with Precision Plus Protein^™^ Unstained Protein Standards (#1610363, Bio-Rad) and its corresponding autoradiography. This experiment was performed twice with identical results.

Since GGOH was detected in mice blood, either from dietary sources or produced by animal PPP (Miriyala et al., 2010; Elsabrouty et al., 2021), we decided to create Δ*Pb*PolK parasite. Importantly, this genomic deletion was deemed possible because previous large-scale studies in *P. berghei* suggested that the targeted gene is non-essential (Janse et al., 2011), as we previously demonstrated with the homologous gene in *P. falciparum* (Crispim et al., 2024). Clones, A and B, were derived from parental *P. berghei* ANKA parasites by replacing the full *Pb*PolK coding sequence by two cassettes expressing resistance to pyrimethamine (hDHFR) or the red fluorescent protein mCherry (Fig 3A). Genomic integration was detected by live fluorescence microscopy (Fig 3B) and by PCR (Fig 3C). Specifically, for the detection of native *Pb*PolK gene by PCR it was employed the primer pair Int-WT-forward and Int-WT-reverse which amplified the expected 1438 bp sequence only in parental parasites. The detection of genomic integration of the recombinant sequence was performed by PCR using the primer pair Int-Δ*Pb*PolK-forward and Int-Δ*Pb*PolK-reverse which amplified a 1233 bp sequence only in transgenic parasites. Finally, we also ensured that transgenic parasites were unable to use exogenous prenols. For this, transgenic parasites (Clone A) and parental parasites were cultured *ex vivo* and radiolabelled with either [^3^H] FOH or [^14^C] isoleucine, employed as a control of protein synthesis (Fig 3D). After incubation, parasites were analysed by sodium dodecyl sulphate–polyacrylamide gel electrophoresis (SDS-PAGE) and autoradiography as described. Only control parasites showed incorporation of [^3^H] FOH into ~8, ~25 and ~50 kDa protein clusters, already known to contain many Ras and Ras-like proteins (Verdaguer et al., 2022a). Both transgenic and parental parasites showed a wide-range incorporation of [^14^C] isoleucine. Results demonstrate that Δ*Pb*PolK parasites do not possess defects on protein synthesis but cannot use exogenous prenols for protein prenylation.

### 2.3 *Pb*PolK knockout parasites are hypersensible to fosmidomycin

**Figure 4:**
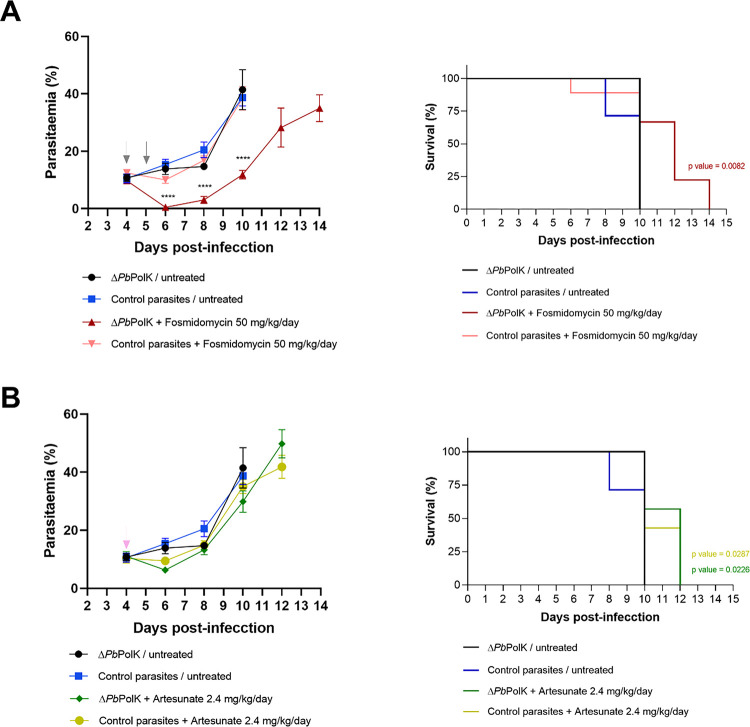
Influence of prenol kinase enzyme on the efficacy of antimalarial drugs. Figure shows the parasitaemia progression and survival of animals infected with either control parasites or Δ*Pb*PolK *P. berghei* parasites. Animals were untreated or treated with suboptimal doses of fosmidomycin (A) or artesunate (B). The graphs represent the mean of 3 independent experiments, with each experimental group containing 3 animals (fosmidomycin-treated animals) and the control groups containing 2 animals (artesunate-treated animals and untreated animals). To compare the parasitaemia progression between strains (e.g. untreated Δ*Pb*PolK vs untreated control parasites; fosmidomycin-treated Δ*Pb*PolK vs fosmidomycin-treated control parasites), the unpaired student’s t-test was employed. *p<0.05, **p<0.01, ***p<0.001, ****p<0.0001. Survival of each group was plotted employing the Kaplan-Meier method. To compare the survival between strains it was employed the log-rank (Mantel-Cox) test (p value indicated in Figure). Log-rank (Mantel-Cox) test was performed to compare survival of untreated Δ*Pb*PolK vs fosmidomycin-treated Δ*Pb*PolK (Graph A); untreated Δ*Pb*PolK vs artesunate-treated Δ*Pb*PolK (Graph B); untreated control parasites vs artesunate-treated control parasites (Graph B). If not indicated, no significant differences were observed. The analyses were performed using the GraphPad Prism^®^ 5.3 program. The two types of pharmacological tests depicted in this figure were conducted simultaneously, and therefore, the untreated controls are the same for both the artesunate-treated and fosmidomycin-treated groups. For clarity, the results of each experiment were presented in separate graphs. Grey and pink arrows indicate the days in which fosmidomycin or artesunate were administered to treated groups, respectively.

After obtaining the transgenic parasites, their antimalarial sensitivity was assessed *in vivo*. Mice were infected with each Δ*Pb*PolK parasites (Clone A) or transgenic *P. berghei* ANKA parasites expressing GFP (control parasites which contains the same selection markers than Δ*Pb*PolK parasites) (Ishino et al., 2006). Four days after infection animals were treated with suboptimal doses of fosmidomycin (50 mg/kg, intraperitoneally). Artesunate (2.4 mg/kg, intraperitoneally) was employed as a control drug, since its activity is apparently not related to PolK expression. Fosmidomycin was administered once daily for two days, and artesunate was administered just once (Wiesner et al., 2002; Osirime et al., 2020). A control group of untreated animals was also included. Untreated Δ*Pb*PolK-infected mice showed no reduction in parasite growth compared to untreated animals infected with the control parasites (Fig 4A & 4B). Control parasites-infected mice treated with fosmidomycin experienced no reduction in parasitaemia and survival time in comparison to untreated animals (Fig 4A). Contrarily to this, animals infected with Δ*Pb*PolK parasites and treated with fosmidomycin experienced a significant parasitaemia decrease during days 5–6 post-infection reaching 1–0.1%. After this, parasitaemia once again increased but on a slower rate than in untreated groups and animals infected with control parasites, therefore significantly extending their survival time. A similar effect was observed when employing Δ*Pb*PolK parasites Clone B, demonstrating that Δ*Pb*PolK high sensitivity to fosmidomycin is not a clone-specific characteristic (S1 Fig). Finally, groups infected with Δ*Pb*PolK and those infected with control parasites, both treated with suboptimal doses of artesunate, experienced a slight reduction in parasitaemia levels and survival time compared to their respective controls. However, no significant differences in parasitaemia levels and survival time were observed between the Δ*Pb*PolK-infected groups and the control parasite-infected groups treated with artesunate (Fig 4B). Therefore, the increased sensitivity to fosmidomycin observed in transgenic parasites is presumably not a general or nonspecific effect that applies to any antimalarial compound.

### 2.4 Geraniol effects the parasitic prenol salvage and favour fosmidomycin efficacy *in vitro.*

*Pf*PolK is a transmembrane enzyme that acts on lipid substrates. Measuring its activity typically requires radiochemical and chromatographic methods, which are costly and time-consuming (Verdaguer et al., 2022b). Therefore, we screened each drug candidate at a single concentration as a proof-of-concept to identify potential inhibitors of the prenol salvage pathway, to be further validated in biological assays.

**Figure 5:**
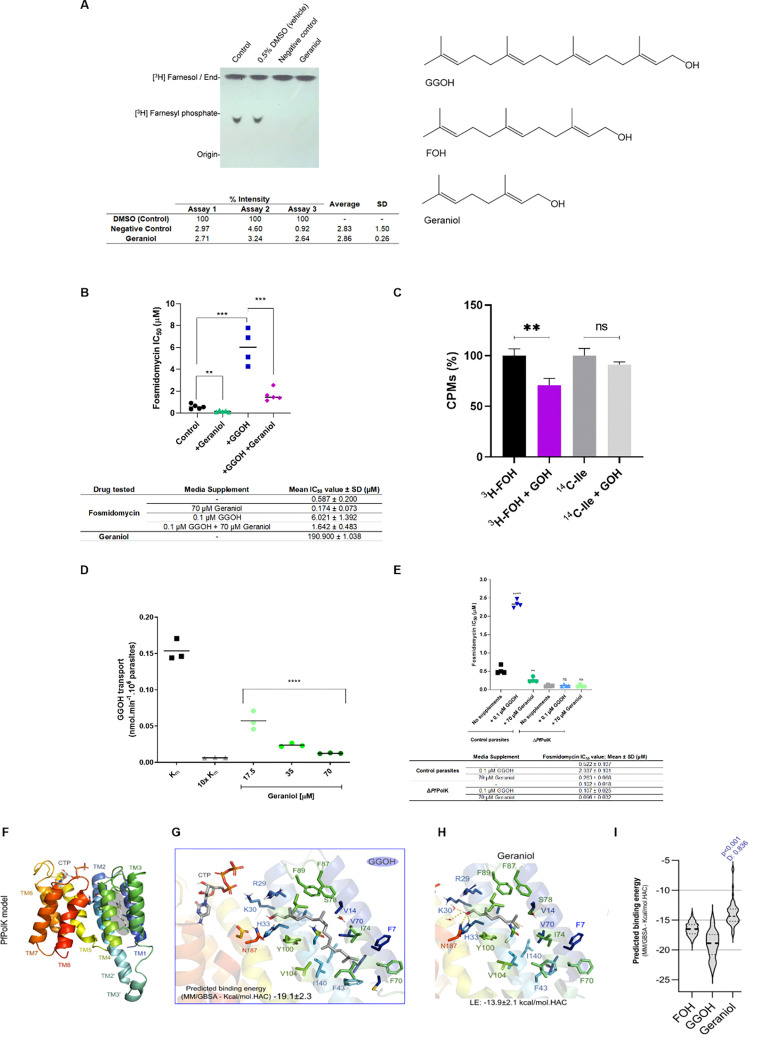
Search for inhibitors of the prenol salvage pathway. (A) Autoradiographs of the PolK enzymatic activity assays using [^3^H] FOH as substrate and chromatographed by TLC. The enzyme source of these assays came from whole extracts of yeast strains transformed with p416-*Pf*PolK. Compounds added to the enzymatic reaction are indicated under the TLC autoradiography image. The TLC retention of FOH and FP is also indicated. Figure also shows the chemical structure of FOH, GGOH, and geraniol (Chemical structures were designed using ChemDraw Software). The figure also displays the quantitative pixel analysis of autoradiographic images. The accompanying table provides data from the analysis of the spot corresponding to FP. The count of opaque pixels was conducted using ImageJ software. The percentage of produced FP was calculated as the ratio of pixels corresponding to FP in enzymatic assays to the positive control (which is considered to represent 100% of produced FP), multiplied by 100, for each experimental replicate. The average and standard deviation (SD) were calculated from three distinct experiments. (B) The graph and its respective table shows the *P. falciparum* 3D7 strain IC_50_ values and dose-response curves of geraniol, and fosmidomycin under the presence of different compounds, as indicated. Statistical analysis was made using one-way ANOVA / Dunnet’s Multiple Comparison Test.*p<0.05, **p<0.01, ***p<0.001 (n = 5). (C) The graph shows the levels of incorporation of [^3^H] FOH into proteins in *P. falciparum* parasites exposed to different 24 h treatments, as indicated. Statistical analysis was made using one-way ANOVA One-way ANOVA / Tukey’s Multiple Comparison Test.*p<0.05, **p<0.01, ***p<0.001. Comparison made to between samples of treated parasites to untreated parasites (n = 3). (D) Figure shows [^3^H] GGOH incorporation levels in *P. falciparum* 3D7 strain cultured under the absence / presence of 70 μM geraniol. This experiment was performed three times. (E) The graph and its respective table show the 72 h IC_50_ value of fosmidomycin under the absence / presence 70 μM geraniol or 0.1 μM GGOH. These experiments were performed with either transgenic *P. falciparum* parasites preserving *Pf*PolK (Control parasites) or lacking it (Δ*Pf*PolK). Statistical analysis was made using one-way ANOVA / Tukey’s Multiple Comparison Test.*p<0.05, **p<0.01, ***p<0.001, ****p<0.0001. Comparison made to control (No supplements) data (n = 4). Panel shows bioinformatic analysis of geraniol. (F) AlphaFold 2 *Pf*PolK model displays eight conserved transmembrane helices (TM1–8, coloured) and a potential prenol binding pocket. (G) This model was used to generate the potential binding of GGOH (blue) by a combination of flexible docking and long molecular dynamics simulations (5×1 μs for each system in explicit solvent and membrane) – modified from Crispim et el., 2024. (H) This model was used to generate the binding mode for geraniol. MD trajectories were utilized to infer the substrates predicted binding energy (kcal/mol.HAC, where HAC – heavy atom count), suggesting from the median values + standard deviation values. (I) MD trajectories were utilized to infer the substrates predicted binding energy (kcal/mol.HAC, where HAC – heavy atom count) depicted as violin-plot distribution for geraniol in comparison to the GGOH substrate. Cumulative MM/GBSA binding energies were compared to the GGOH using Kolmogorov-Smirnov test, whose p-values and D-values are explicitly shown above their respective distributions.

A collection of terpene compounds, chemically similar to PolK lipid substrate, was chosen for testing. This collection includes acyclic monoterpenes, cyclic terpenes, terpene-like unsaturated molecules, and both natural and synthetic sesquiterpenes (nerol, nerolidol, geraniol, limonene, geranylgeranyl acetone, linalool, citral, citronellol, geranic acid, perillyl alcohol, perilic acid, and 2,7-dimethyl-1-octanol; see Figs 5A and S2). For this, we employed as expression system *S. cerevisiae*, which has been demonstrated not to possess PolK enzymes (Voshall et al., 2024), as described elsewhere (Crispim et al., 2024). Specifically, The W303–1A strain of *S. cerevisiae* complemented with the p416-*Pf*PolK plasmid was grown in SD-Uracil medium and used for enzymatic assays. We incubated yeast extracts with [^3^H] FOH plus CTP. In some cases, CTP was omitted (kinase activity control), or it was added the drug-vehicle as control (0.5% DMSO). Also, some assays were done including the previously identified candidate inhibitors at a final concentration of 200 μM, i.e., x100 fold the concentration of [^3^H] FOH. In most cases, the reactions produced radiolabelled compounds with chromatographic retention compatible with FP (Fig 5A). However, the formation of FP was strongly inhibited in assays that included geraniol (2.86 ± 0.26% FP band intensity relative to control assays without the addition of any drug). These values are similar to those of the Negative Control (without addition CTP) which still phosphorylated a small amount of FOH, most likely due to the presence of CTP traces in the yeast extract (2.83 ± 1.5% FP band intensity compared to control assays without the addition of any drug). Considering this, we presumed that geraniol may act as inhibitor of PolK and thus, we further studied its *in vitro* effects on *P. falciparum* parasites.

Geraniol demonstrated low toxicity for the parasite, as indicated by its IC_50_ value being in the high micromolar scale (Fig 5B). These findings provide limited insight into its pharmacological potential, especially considering that the prenol salvage pathway is not essential for the survival of malaria parasites (Pavan et al., 2018; Crispim et al., 2024). A concentration of 70 μM of geraniol was found to be slightly toxic at 72 h for the parasite and was therefore selected to further explore their biological effects (S3 Fig). Subsequently, we evaluated the antiparasitic effects of fosmidomycin on *P. falciparum* at 72 h, in the presence or absence of various combinations of 70 μM geraniol, and 0.1 μM GGOH (Fig 5B). In these assays, the average IC_50_ value for fosmidomycin alone was 0.587 ± 0.2 μM. As anticipated, the addition of GGOH increased the IC_50_ value of fosmidomycin significantly, by 10.26-fold (IC_50_ = 6.021 ± 1.392 μM). The introduction of geraniol reduced 3.37-fold the antimalarial effect of fosmidomycin (IC_50_ = 0.174 ± 0.072 μM μM for geraniol addition). Crucially, adding geraniol along with GGOH significantly reduced the GGOH rescue effect, making the IC_50_ value of fosmidomycin approximately 3.67-fold lower to that calculated for the addition of just GGOH (IC_50_ = 1.642 ± 0.483 μM μM for geraniol plus GGOH addition). As a control, we conducted this assay using citronellol, nerol, and 2,7-dimethyl-1-octanol. By using these compounds, we observed negligible or absence of this blockade of GGOH-rescue of fosmidomycin effects (Fig S4). This suggests that the biological activities of geraniol are not common to all terpene and terpene-like compounds.

We further studied the effects of geraniol on prenol metabolism. For this, *P. falciparum* parasites were cultured with [^3^H] FOH or [^14^C] isoleicine (Fig 5C). Parasites were also exposed to 70 μM geraniol. After 24 h, radioincorporation into TCA-precipitated proteins was assessed. In comparison to geraniol-treated parasites, control parasites showed more incorporation of [^3^H] FOH into proteins. However, geraniol did not affect [^14^C] isoleucine incorporation into proteins, indicating that geraniol does not affects protein synthesis but the utilization of exogenous FOH for protein prenylation. To deepen the understanding of geraniol mechanism of action, it was decided to assess their effects on the incorporation of GGOH into parasites (Fig 5D; see another experiment in Fig S5 Fig). For this, we employed the protocol previously employed to assess the kinetics of [^3^H] GGOH transport in *P. falciparum* parasites (Verdaguer et al., 2022a). Importantly, [^3^H] GGOH exhibits a saturable profile of temporal incorporation that conforms to an exponential decay function, indicating the involvement of a carrier that facilitates an average V_max_ of 0.134 nmol.min^−1^ and a K_m_ value of 0.315 mM. Near-linear incorporation occurs during the first 30 min (Verdaguer et al., 2022a). Therefore, our assays were conducted to measure [^3^H] GGOH transport levels in the presence or absence of different concentrations of geraniol for 30 min. The results showed that geraniol inhibits [^3^H] GGOH transport in a dose-responsive manner, reducing it up to 8.7-fold.

To unequivocally assess if the previously mentioned biological effects of geraniol are related to the prenol salvage pathway. For this purpose, we utilized the inducible *Pf*PolK knockout strain of *P. falciparum* (Crispim et al., 2024), in which the *Pf*PolK gene excision was either not induced (control parasites) or induced (Δ*Pf*PolK). Transgenic *P. falciparum* parasites, either expressing or lacking *Pf*PolK, were used to calculate the IC_50_ value of fosmidomycin under the absence or presence of 70 μM geraniol, or 0.1 μM GGOH, a control to indirectly assess the absence or presence of *Pf*PolK (Fig 5E). Control parasites exhibited an IC_50_ value for fosmidomycin of 0.522 ± 0.107 μM. This value increased 4.5-fold when GGOH was added to the culture medium (IC_50_ 2.337 ± 0.101 μM) and significantly reduced approximately 2-fold due to the addition of geraniol (IC_50_ of 0.263 ± 0.068 μM). In comparison, Δ*Pf*PolK parasites showed a 5-fold reduced IC_50_ value for fosmidomycin (0.102 ± 0.018 μM), a reduction likely related to small amounts of GGOH in bovine media supplements, as previously discussed (Crispim et al., 2024). Notably, the IC_50_ value of fosmidomycin in Δ*Pf*PolK parasites was not significantly affected by the addition of geraniol or GGOH. Therefore, it is concluded that geraniol negatively affect fosmidomycin efficacy through mechanisms closely related to the prenol salvage pathway.

Finally, we evaluated the potential inhibition mechanism of geraniol through bioinformatic approaches using the previously generated *Pf*PolK model (Crispim et al., 2024). Briefly, *Pf*PolK model displays eight conserved transmembrane helices (TM1–8) and a potential prenol binding pocket (Fig 5F). The potential binding of *Pf*PolK-GGOH was calculated through flexible docking followed by molecular dynamics (MD) simulations (Fig 5G) and used as basis to model how the geraniol would bind to *Pf*PolK (Fig 5H). Molecular dynamics (MD) trajectories were analysed to estimate the predicted substrate binding energy using MM/GBSA (Molecular Mechanics/Generalized Born Surface Area). The comparison between the predicted binding energy of geraniol and GGOH shows comparable distributions, suggesting that geraniol may compete for substrate binding to *Pf*PolK (Fig 5I). Despite these remarks, it should be noted that all these computational predictions need additional experimental validation.

### 2.5 Geraniol slightly enhances fosmidomycin efficacy *in vivo*.

**Figure 6:**
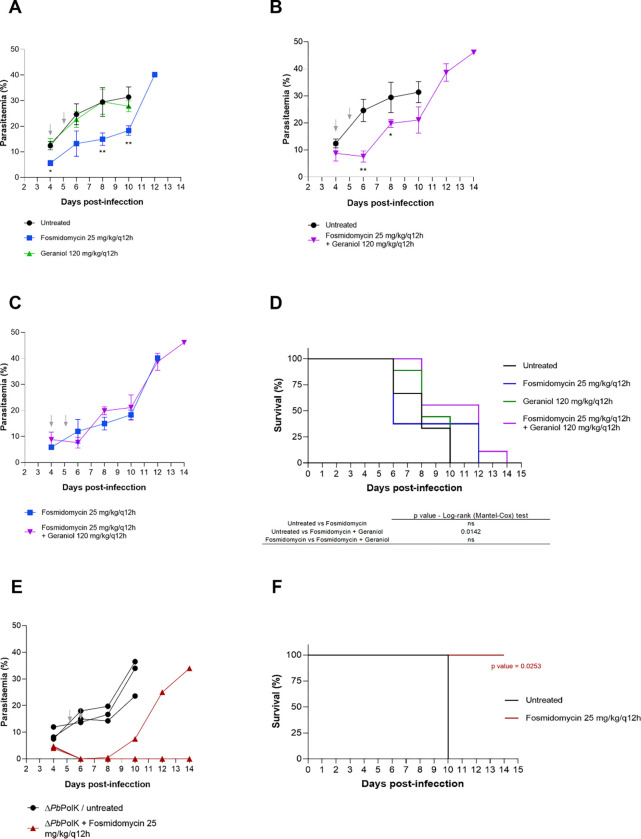
Geraniol improves the efficacy of fosmidomycin *in vivo*. The figure displays the parasitaemia progression and survival of *P. berghei* ANKA (control parasites) infected animals treated with (A) fosmidomycin or geraniol, or (B) a combination of both compounds. A control group of untreated animals was also included. In (C), the comparison between treatments with only fosmidomycin or fosmidomycin combined with geraniol is shown. The graphs represent the mean of 3 independent experiments, with each experimental group containing 3 animals (treated animals) and the control groups containing 3 animals (untreated animals). The figure (D) displays the survival of *P. berghei* ANKA infected animals untreated or treated with fosmidomycin, geraniol or a combination of both compounds. Figure (E) depicts the progression of parasitaemia in *Pb*PolK knockout parasites, either treated or untreated with fosmidomycin. The graph represents the mean from a single experiment, with each group comprising 3 animals. Figure (I) shows the survival of animals infected with *PbPolK* knockout parasites, either untreated or treated with fosmidomycin according to the described drug regimen. In all experiments depicted in this figure, an unpaired Student’s t-test was employed to compare the progression of parasitaemia between groups (e.g., untreated vs. fosmidomycin-treated; fosmidomycin-treated vs. fosmidomycin plus geraniol-treated). *p<0.05, **p<0.01, ***p<0.001, ****p<0.0001. Survival of each group was plotted employing the Kaplan-Meier method. To compare the survival between groups it was employed the Log-rank (Mantel-Cox) test. *p<0.05, **p<0.01, ***p<0.001, ****p<0.0001. If not indicated, no significant differences were observed. The analyses were performed using the GraphPad PRISM^®^ 5.3 program.

As observed, geraniol inhibits PolK and enhances the efficacy of fosmidomycin even in the presence of GGOH, making it an interesting candidate for *in vivo* testing in mice. As will be better discussed later, geraniol has a short half-life. Therefore, compared to the previous experiment involving transgenic parasites, in which a single daily dose of 50 mg/kg was administered, we decided to increase the frequency of drug administration in this study. Specifically, the fosmidomycin regimen was adjusted to two doses of 25 mg/kg every 12 hours (q12h), and geraniol was administered orally at a well-tolerated high dose (120 mg/kg/q12h) (Fig. 6A). The selected dose of geraniol corresponds to the maximum non-toxic level determined in earlier studies (Pavan et al., 2018), while the fosmidomycin dosage adjustment was based on its pharmacokinetics, aiming to enhance treatment efficacy (Na-Bangchang et al., 2007; Murakawa et al., 1982; Walker, 2018).

In all cases, animals were infected with wild-type *P. berghei* ANKA parasites and, four days later, received the respective treatments. Compared to untreated animals, those treated with fosmidomycin showed a reduction in parasitaemia on specific days, although this did not result in extended survival. Mice treated with the combination of fosmidomycin and geraniol exhibited reduced parasitaemia and prolonged survival compared to untreated controls. However, the log-rank (Mantel-Cox) test revealed no statistically significant difference between the fosmidomycin-only and fosmidomycin-plus-geraniol groups.

We also conducted an experiment using two groups of mice infected with *PbPolK* knockout parasites (Clone B), which were either treated or not treated with fosmidomycin under the same regimen (n = 3 animals per group). These groups were included to evaluate how the complete absence of *Pb*PolK would influence the response to the adjusted fosmidomycin treatment (Fig. 6B). In this case, two out of the three treated animals were cured, with no detectable parasites until the end of the experiment (day 14 post-infection). In the third animal, parasitaemia reappeared after treatment but progressed slowly and did not result in death before the end of the experiment.

Altogether, these results indicate that geraniol does not possess intrinsic antimalarial activity and does not significantly enhance the efficacy of fosmidomycin *in vivo* under our treatment regimen. However, the comparison between Δ*Pb*PolK-infected mice and wild-type-infected mice strongly suggests that, under a q12h/2-day treatment schedule of fosmidomycin, an effective PolK inhibitor may potentiate fosmidomycin’s efficacy to achieve a curative outcome *in vivo*.

### 2.6 Proteomic analysis of the parasitic prenol salvage pathway

**Figure 7:**
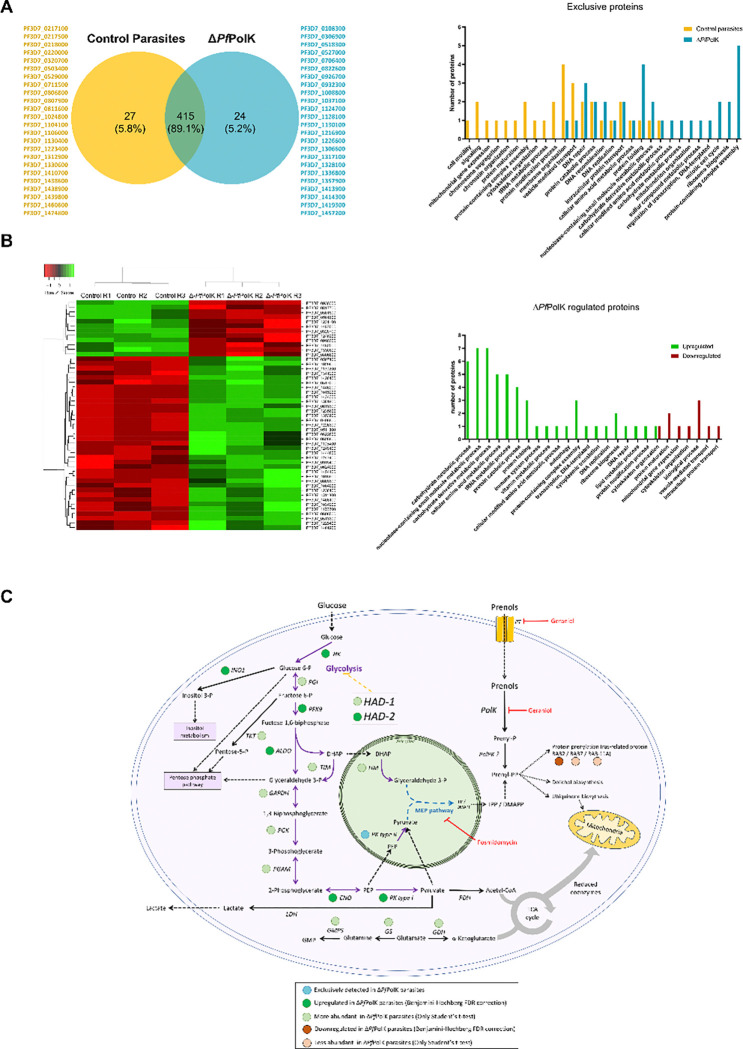
Comparative proteomic and metabolic regulation in Δ*Pf*PolK parasites. A. Panel shows a Venn diagram of the proteomic profiles comparing transgenic *P. falciparum* parasites preserving *Pf*PolK (Control parasites) versus knockout parasites (Δ*Pf*PolK). The excision of the floxed *Pf*PolK-loxP in Δ*Pf*PolK parasites was carried out by adding 50 nM rapamycin or DMSO (used as a vehicle control) to synchronized ring-stage cultures. The cells were treated for 24 h, followed by a washout step. Subsequently, parasites were cultured again without rapamycin for an additional three parasitic cycles before collecting samples. Figure also contains a table detailing protein name, PlasmoDB code and GO term. B. Panel presents a heat map illustrating the differential protein expression between control and *Pf*PolK knockout. The figure also contains a table detailing protein name, PlasmoDB code and GO term. Yellow indicates proteins exclusively found in control parasites; Blue indicates proteins exclusively found in Δ*Pf*PolK parasites; Green indicates upregulated proteins of Δ*Pf*PolK parasites, in comparison to control parasites; Red indicates downregulated proteins of Δ*Pf*PolK parasites, in comparison to control parasites. These experiments were performed with 3 biological replicates for each sample (parasites preserving or not *Pf*PolK). C. Figure depicts the glycolysis / MEP pathways and shows results obtained through proteomics and the targets of fosmidomycin and geraniol. Continuous arrows represent a single enzymatic step, while discontinuous arrows indicate multiple steps or the crossing of metabolites through membranes. Purple arrows denote the glycolytic process, and blue arrows refer to the MEP pathway. Red blunt-end lines highlight drug inhibition, and yellow dotted blunt-end lines indicate HAD-mediated dephosphorylation of glycolytic intermediates. Colored circles represent protein regulation, as shown in the legend. The analysis of protein abundance between the transgenic *P. falciparum* parasites preserving *Pf*PolK (Control parasites) versus knockout parasites (Δ*Pf*PolK) was performed using two different statistical methodologies, as indicated in figure legend. The more restrictive method involved an unpaired Student’s t-test of LFQ values and correcting for Benjamini-Hochberg based FDR. Proteins with an FDR < 0.05 were considered up/downregulated proteins. Alternatively, the less restrictive method involved an only Student’s unpaired t-test of LFQ values for group comparison, where proteins with a p-value < 0.05 were considered more/less abundant proteins. The symbol “?” denotes suspected processes that are not yet well characterized. Italicized letters represent enzymes. Abbreviations not cited in text: Hexokinase (HK), glucose-6-phosphate isomerase (PGI), phosphofructokinase (PFK9), fructose-bisphosphate aldolase (ALDO), triose-phosphate isomerase (TIM), glyceraldehyde 3-phosphate dehydrogenase (GAPDH), phosphoglycerate kinase (PGK), 2,3-bisphosphoglycerate-dependent phosphoglycerate mutase (PGAM), enolase (ENO), pyruvate kinase (PK), lactate dehydrogenase (LDH), pyruvate dehydrogenase (PDH), glutamate dehydrogenase (GDH), glutamine synthetase (GS), GMP synthetase (glutamine-hydrolysing) (GMPS), Tricarboxylic acid (TCA), Transketolase (TKT).

We decided to explore the biological role of the parasitic prenol salvage pathway through proteomics. To this end, we compared proteomic profiles between transgenic *P. falciparum* parasites preserving the *Pf*PolK gene (control) to parasites in which the gene was excised through rapamycin treatment, as described in methodological procedures and Fig.7 legend (Fig 7). Proteomic analysis was performed with three biological replicates of each sample (parasites preserving or not *Pf*PolK) and 442 proteins were identified in control parasites compared to 439 in *Pf*PolK knockout parasites (Fig 7A). Of these, 24 proteins were only present in parasites lacking *Pf*PolK compared to the control, which had 27 exclusive proteins found (Fig 7A). Importantly, the apicoplast pyruvate kinase II (PF3D7_1037100) was exclusively detected in parasites lacking *Pf*PolK, and not in control parasites (Swift et al., 2020). An unpaired Student’s t-test followed by Benjamini-Hochberg FDR correction (FDR < 0.05) was applied to identify up and downregulated proteins between groups. Molecular deletion of *Pf*PolK upregulated the expression of 37 proteins and downregulated 12, compared to control parasites (Fig 7B). Most regulated proteins are enzymes (tables showing all detected proteins are in Supplementary Information Spreadsheet 1), especially those related to the following GO terms: carbohydrate metabolic process (GO:0005975), nucleobase-containing small molecule metabolic process (GO:0055086), cellular amino acid metabolic process (GO:0006520), carbohydrate derivative metabolic process (GO:1901135), tRNA metabolic process (GO:0006399), protein catabolic process (GO:0030163). It is of special interest to comment that the excision of *Pf*PolK upregulated the expression of proliferating cell nuclear antigen 1 (PCNA, PF3D7_1361900), several tRNA ligases (e.g.: leucine--tRNA ligase, PF3D7_0622800; cysteine--tRNA ligase, PF3D7_1015200), as well as several enzymes related to oxidative stress defense (e.g.: glutathione S-transferase, PF3D7_1419300 thioredoxin 1, PF3D7_1457200) and proteostasis (e.g.: heat shock protein 110, PF3D7_0708800; Hsp70/Hsp90 organizing protein, PF3D7_1434300), together with some enzymes related to protein catabolism (e.g.: plasmepsin III, PF3D7_1408100; aminopeptidase P, PF3D7_1454400). Furthermore, the excision of *Pf*PolK make no detectable or downregulated Ras-related protein Rab-2 (PF3D7_1231100) and merozoite surface protein 1 and 2, respectively (PF3D7_0930300 and PF3D7_0206800). These are prenylated/glycosylated proteins which may been affected of some dysregulation of the isoprenoid metabolism. The reason for the regulation of most proteins remains unexplained at this time, except for those enzymes involved in carbohydrate metabolic processes. Parasites lacking *Pf*PolK showed an upregulation of HAD-2 (PF3D7_1226300) along with several glycolytic enzymes or glucose metabolism-related enzymes, including hexokinase (PF3D7_0624000), fructose-bisphosphate aldolase (PF3D7_1444800), enolase (PF3D7_1015900), and the glycolytic pyruvate kinase type I (PF3D7_0626800) (Swift et al., 2020) and inositol-3-phosphate synthase (PF3D7_0511800).

The comparison between groups using only Student’s t-test led to the identification of what we refer to here as more/less abundant proteins. This analysis was performed to capture more subtle regulatory changes, complementing the more stringent FDR-based approach. The results were largely consistent with the previously discussed findings: it was found an increased expression of HAD-1 (PF3D7_1033400) and other glycolytic enzymes such as glucose-6-phosphate isomerase (PF3D7_1436000), triosephosphate isomerase (PF3D7_1439900), glyceraldehyde-3-phosphate dehydrogenase (PF3D7_1462800), phosphoglycerate kinase (PF3D7_0922500), and phosphoglycerate mutase (PF3D7_1120100) as well as transketolase (PF3D7_0610800), involved in the pentose phosphate pathway. Interestingly, it was also observed an increased expression of the glutamate-consuming enzymes glutamate dehydrogenase (PF3D7_1416500), glutamine synthetase (PF3D7_0922600), and GMP synthetase (glutamine-hydrolysing) (PF3D7_1012600). Interestingly, it was observed a decreased expression to ras-related protein RAB7 (PF3D7_0903200) and ras-related protein Rab-11A (PF3D7_1320600).

## 3. DISCUSSION

In clinical trials, the use of fosmidomycin, either alone or in combination with other drugs, has been unsuccessful due to its poor antimalarial activity and recrudescence. Importantly, no mutations related to resistance have been detected in these trials (Lanaspa et al., 2012; Fernandes et al., 2015; Mombo-Ngoma et al., 2018), suggesting that the failure may be attributed to intrinsic mechanisms in the parasite (Guggisberg et al., 2016). As mentioned, we previously demonstrated that human plasma contains lipidic factors, most likely GGOH/GGPP, that can diminish the antimalarial activity of fosmidomycin in *P. falciparum* parasites. Further studies identified a gene in malaria parasites encoding a PolK enzyme, whose inhibition makes *P. falciparum* parasites more sensitive to fosmidomycin and unable to utilize prenols (Crispim et al., 2024). All these data make us hypothesize that, despite individual variability and dietary influence, blood levels of certain isoprenoid precursors may reach concentrations high enough to reduce fosmidomycin’s effectiveness *in vivo*. To clarify this, we aimed to evaluate our hypothesis using the rodent parasite *P. berghei*.

First, we ensured that the mice used in our facilities are naturally exposed to dietary sources of prenols and confirmed that mouse plasma contains GGOH, which may originate from dietary intake and/or endogenous biosynthesis via the MVA pathway (Miriyala et al., 2010; Elsabrouty et al., 2021; Verdaguer et al., 2022b). Then, we evaluated whether PolK deletion affects the efficacy of drugs that do not directly target isoprenoid metabolism in *P. falciparum* parasites. Although only a limited set of antimalarial drugs was tested, it became evident that the increased sensitivity to fosmidomycin appears to be highly specific. In fact, among the drugs analyzed, only quinine and mefloquine exhibited a significantly increased efficacy in *P. falciparum* parasites lacking PolK, although this effect was not as pronounced as with fosmidomycin. The increased sensitivity to quinine and mefloquine is not fully understood, especially since its exact molecular target remains unknown. However, it is likely linked to the well-documented inhibitory effects of both drugs on glycolysis and carbohydrate metabolism, which are also associated with PolK expression, as discussed below (Speck, 1945; Marshall, 1948; Moulder, 1948). In any case, this finding suggests that the prenol salvage pathway plays a significant role in regulating the overall metabolism of malaria parasites and may indirectly influence the efficacy of some drug classes that are not directly related to isoprenoid metabolism. Thus, the prenol salvage pathway warrants further investigation, not only in the context of MEP pathway-targeting drugs but also for the development of new antimalarial candidates and a deeper understanding of the mechanisms underlying drug resistance. For now, this small drug screening has helped us identify several compounds unrelated to PolK expression, making them suitable as controls in *in vivo* assays using *P. berghei* knockout parasites. Among these, we selected artesunate as the control drug, as explained further below.

Once the feasibility of our *in vivo* model was assessed, we generated *P. berghei* parasite knockout for *Pb*PolK. These parasites proved to be viable and showed no differences in disease progression compared to control parasites when no treatment was administered. However, Δ*Pb*PolK parasites did not incorporate radiolabeled FOH into proteins and caused a form of murine malaria that responded more favourably to fosmidomycin treatment, in comparison to animals infected with control parasites. In fact, mice infected with ΔPbPolK parasites and treated with 25 mg/kg fosmidomycin every 12 hours for only two days achieved a curative outcome *in vivo* in most cases—whereas the same treatment was completely ineffective against wild-type parasites. This differentiated response to malaria treatment appears to be specific to fosmidomycin, as no differences in response between the strains were observed when using the antimalarial artesunate. These findings bolster the concept that the prenol salvage pathway is not a critical metabolic path. However, clearly, they highlight its importance in the context of fosmidomycin exposure, as it significantly limits the drug antimalarial efficacy. Therefore, the primary conclusion of this research is that the prenol salvage pathway represents as a new drug target to enhance the effectiveness MEP inhibitors. Moreover, our results demonstrate that the scavenging of host-derived isoprenoids may serve as a natural resistance mechanism against MEP inhibitors in the blood stages of malaria parasites, revealing a potential therapeutic vulnerability. Similarly, host isoprenoid scavenging has been shown to reduce the efficacy of fosmidomycin against *P. berghei* liver stages and bisphosphonates against *Toxoplasma gondii* in *in vivo* models (Li et al., 2013; Walker et al., 2024). In these cases, the reliance on host isoprenoids was more evident, as these parasites reside within host cells that possess an active MVA pathway. Importantly, while this study focused on fosmidomycin as a specific inhibitor of the MEP pathway in malaria parasites, other MEP-targeting drugs, such as ribosome inhibitors (e.g., clindamycin), may also benefit from PolK disruption.

Based on last commented results, we conducted a search for a PolK inhibitor using biochemical and bioinformatic approaches, alongside *in vitro* assays in various *P. falciparum* strains. Our research concentrated on specific natural terpenes, which are known to disrupt isoprenoid metabolism in malaria parasites and other cellular systems through mechanisms that are not yet fully understood (Elson et al., 1999; Gabriel et al., 2018; Neighbors, 2018). Besides several compounds, only geraniol demonstrated the ability to inhibit recombinant *Pf*PolK activity and potentiated the *in vitro* the antimalarial effect of fosmidomycin in *P. falciparum*, even under the presence of GGOH. Molecular docking with the AlphaFold-retrieved model suggested that geraniol possibly inhibits PolK in a competitive manner with the lipid substrate, which seems plausible due to its significant chemical similarity to FOH/GGOH. Further experiments revealed that geraniol reduce [^3^H] GGOH incorporation into *P. falciparum* parasites and [^3^H] FOH incorporation into proteins. This effect could be attributed to geraniol inhibition of PolK but also to other reasons such as the inhibition of prenol transport, prenyl transferases or even the still unidentified PolPK enzyme. The concentration required to inhibit the utilization of FOH/GGOH by parasites are relatively high, at 70 μM. This indicates that geraniol is likely a weak inhibitor of the prenol salvage pathway. However, geraniol appears to exhibit a significant level of specificity for inhibiting the prenol salvage pathway. This statement is supported by experiments conducted with transgenic *P. falciparum* parasites, preserving or not PolK, which demonstrated that the potentiation of fosmidomycin effects by geraniol depends on the presence of a functional PolK enzyme. Importantly, our results do not exclude the possibility that geraniol, closely related to the intermediates of isoprenoid biosynthesis, could act as a substrate for PolK and thus being phosphorylated. In this hypothetical scenario, the fact that geraniol does not rescue malaria parasites from fosmidomycin would need to be explained by other factors, such as geranyl-P not being a suitable substrate or even inhibiting the still unidentified PolPK.

The *in vivo* administration of geraniol at high doses did not result in a significant reduction in parasitaemia neither improved the efficacy of fosmidomycin when both compounds were administered together. The limited success of geraniol in enhancing fosmidomycin’s efficacy *in vivo* may be attributed to its short half-life in blood, which has been calculated to be 12.5 ± 1.5 minutes in rats (Pavan et al., 2018; Mączka et al., 2020). Possibly for this reason, in studies investigating the effects of geraniol on other diseases in mouse models, some authors administered it mixed with the animals’ chow, which may have prolonged its systemic availability (Galle et al., 2014). However, we chose not to administer geraniol this way, as it does not reflect clinically relevant conditions for human use. Additionally, due to its strong odor, we considered that mixing geraniol with food could negatively affect food intake—particularly the consumption of GGOH—thus introducing several difficult-to-control variables into the experiment. Therefore, we emphasize that geraniol should not be considered a standalone treatment but rather a research tool and a chemical starting point for developing more effective PolK inhibitors to enhance the efficacy of fosmidomycin. Supporting this proposal, geraniol is classified as Generally Recognized As Safe (GRAS) by the U.S. Food and Drug Administration due to its low toxicity, with a lethal dose 50 (LD_50_) in rats estimated at approximately 3600 mg/kg (Lewis, 2004). Regarding its bioavailability, studies in rats have shown that geraniol has an absolute oral absorption rate of 92% in the gut and can also reach the cerebrospinal fluid (Pavan et al., 2018).

Finally, the biological role of the prenol salvage pathway in malaria parasites was further investigated through proteomics. Surprisingly, proteomic analyses revealed a ambiguous effect: an upregulation of HAD enzymes, along with increased expression of glycolytic enzymes. At this point, it must be acknowledged that the higher expression of HAD enzymes following PolK excision is not well understood and requires further investigation. However, in broader terms, these results suggest that PolK plays a regulatory role in sugar and lipid metabolism. This regulatory effect may occur through two alternative pathways. (I) **Regulation of the glycolytic pathway:** Following PolK excision, there is an overexpression of glycolytic enzymes, which may serve as tentative to enhance MEP pathway activity and compensate for the lack of exogenous isoprenoid utilization. If this is the case, the overexpression of HAD enzymes could function as a mechanism to control excessive glycolytic activity. (II) **Regulation of HAD enzymes**: If PolK disruption directly promotes HAD expression, one possible explanation is that intracellular GGOH and/or FOH act as signals indicating sufficient isoprenoid availability, thereby triggering MEP downregulation through HAD-mediated dephosphorylation of glycolytic intermediates. If so, the observed upregulation of glycolytic enzymes could represent a compensatory response to counteract excessive dephosphorylation and maintain normal growth rates. Supporting this second hypothesis, previous studies have shown that fosmidomycin-resistant parasites exhibit genetic changes in HAD proteins, leading to reduced growth and, presumably, increased glycolytic activity (Guggisberg et al., 2018). Once fosmidomycin was withdrawn, these parasites acquired mutations in glycolytic enzymes, which restored glycolysis to physiological levels while simultaneously recovering parasitic growth and fosmidomycin sensitivity. Similarly to this hypothesis, in animals, GGOH has been shown to promote the proteolytic degradation of MVA pathway enzymes, thereby reducing isoprenoid biosynthesis (Schumacher et al., 2015).

In conclusion, our findings highlight the role of the PolK and the whole prenol salvage pathway in modulating metabolic plasticity, allowing the utilization of host-derived GGOH, and limiting fosmidomycin efficacy *in vivo*. Furthermore, it is described an inhibitor able to potentiate fosmidomycin antimalarial efficacy and partially block the utilization of exogenous GGOH by parasites *in vitro*. Further research will be necessary to better understand how PolK modulates general carbon and lipid metabolism in the parasite and to identify more effective PolK inhibitors. Our results pave the way for further investigation into the development of novel combination therapies for malaria treatment, targeting both the MEP pathway and utilization of host isoprenoids. Finally, we would like to emphasize that the importance of this study is not limited to malaria, but also concerns several other diseases. In fact, GGOH from dietary sources has been suggested to limit the antineoplastic activity of statins, and MEP/MVA inhibitors are being studied for the treatment of several infectious diseases besides malaria (de Wolf et al, 2017; Jawad et al, 2022; Verdaguer et al., 2022b). Therefore, the biomedical importance of the prenol salvage pathway may apply to several diseases related to isoprenoid metabolism.

## 4. CONCLUSIONS

This study highlights the prenol salvage pathway as a key regulator of metabolic homeostasis in *Plasmodium*, enabling the parasite to utilize host-derived isoprenoids and thereby reducing the efficacy of fosmidomycin. These findings provide a rationale for combining MEP pathway inhibitors with PolK inhibitors to improve malaria treatment and combat drug resistance. Finally, this study underscores the biomedical significance of the prenol salvage pathway, which may be relevant to a variety of other diseases.

## 5. Materials and methods

### 5.1 Ethics statement

Mice were handled in accordance with the ethical principles of animal experimentation adopted by the Brazilian Society of Laboratory Animal Sciences, and the project as a whole was approved by the The Animal Ethics Committee (CEUA) of the Institute of Biomedical Sciences of the University of São Paulo (CEUA license 9606020323 and 2420280823).

### 5.2 Reagents, stock solutions and plasmids

Albumax I, RPMI-1640, and fosmidomycin sodium salt hydrate were purchased from Thermo Fisher Scientific^®^ (Leicestershire, UK). [1-(n)-^3^H] FOH (20 Ci/mmol; 1 mCi/mL), [1-(n)-^3^H], GGOH (50 Ci/mmol; 1 mCi/mL), L-[4,5-^14^C (U)] isoleucine (200–300 mCi/mmol; 0.1 mCi/mL), D-[U-^14^C]glucose (310 mCi/mmol; 0.2 mCi/mL) were purchased from American Radiolabeled Chemicals^®^ (St. Louis, USA). SYBR Green I^®^ nucleic acid gel stain was purchased from Thermo Fisher Scientific^®^ (Waltham, Massachusetts, EUA). Prenyl phosphates were obtained by mild acid treatment of the respective commercial pyrophosphates (Sigma) (Ohnuma et al, 1996). The rest of reagents not commented here were purchased from Sigma^®^ (St. Louis, Missouri USA) or in specific companies cited in text. For *in vitro* assays in *P. falciparum* parasites, sterile stock solutions were prepared 10 mM for fosmidomycin sodium salt hydrate in water, 200 mM or 2 M for all terpene compouds in ethanol. For enzymatic assays, all drugs were prepared at 40 mM in DMSO. For *in vivo* use, sterile stock solutions were prepared as follows: fosmidomycin sodium salt hydrate was dissolved at 10 mg/ mL in PBS for intraperitoneal administration; artesunate was dissolved at 0,24 mg/ mL in water for oral administration; geraniol was prepared 24 mg / mL emulsion in glycerol. The plasmid p416-GPD-*Pf*PolK for yeast recombinant expression of *Pf*PolK was created previously in our group (Crispim et al., 2024) and the original DcOmCherry plasmid used for deletion of PolK in *P. berghei* was a gift of Prof. Daniel Youssef Bargieri (Department of Parasitology, Institute of Biomedical Sciences of the University of São Paulo, São Paulo, Brazil) (Bargieri et al., 2016). Geranic acid with 85% purity was purchased from Sigma and purified as described elsewhere (Zakrewsky et al., 2016). Briefly, geranic acid was recrystallized five times at −70 °C in acetone, in a 500-mL round bottom flask. The crystallized geranic acid was dried under vacuum to remove any traces of solvent.

### 5.3 Parasite and yeast strains

In this study, we used the *P. falciparum* 3D7 strain, and a *P. falciparum* transgenic strain for the inducible excision of *Pf*PolK derived from the NF54 strain (Δ*Pf*PolK; PfNF54_070015200) which was previously created by our group (Crispim et al., 2024). Briefly, Δ*Pf*PolK was created using Cas9-assisted genome editing, all 612 bp of the native *Pf*PolK open reading frame were replaced by a recodonized sequence flanked by two loxP sites. Rapamycin-induced site-specific excision by recombination between the loxP sites.

Finally, we employed the *P. berghei* ANKA strain (referred as wild-type parasites) used for Δ*Pb*PolK generation and transgenic *P. berghei* parasites expressing GFP, also referred as control parasites (Ishino et al., 2006), containing the same selection markers as Δ*Pb*PolK, were provided by Prof. Daniel Youssef Bargieri (Department of Parasitology, Institute of Biomedical Sciences of the University of São Paulo, São Paulo, Brazil) (Bargieri et al., 2016). The W303–1A strain of *S. cerevisiae* (MATa/MATα {leu2–3,112 trp1–1 can1–100 ura3–1 ade2–1 his3–11,15} [phi^+^]) was a gift of Dr. Agustín Hernández (Betternostics S.L., Noain, Navarre, Spain).

### 5.4 Preparation of mouse chow extracts

For this research, we needed to prepare an extract of the unsaponifiable lipids from the same mouse chow that was used to feed the mice in the *in vivo* experiments. (Nuvilab CR-1; Quimtia, Colombo, Paraná State, Brazil; Ingredients: whole Corn, Soybean Meal, Wheat Bran, Vitamin A, D3, B1, B11, B12, B6, and E). For this, mouse chow was first liquefied with 100 mL of water and filtered using filter paper (de Wolf et al., 2017; Jawad et al., 2022). Following this, the material was transferred to a separating funnel and extracted with 60 mL methanol and 30 mL chloroform. The lower lipid phase was evaporated, and the residue was dissolved in 25 mL ethanol. This extract was treated with 25 mL of 5 M potassium hydroxide at 56°C for 1 h. After cooling and neutralization with 25 mL of 5 M hydrochloric acid, the solution was extracted with 120 mL n-hexane, 30 mL water, and 30 mL ethanol. The upper organic phase was evaporated and dissolved in at 10 mg/ mL ethanol and employed as stock solution.

### 5.5 Handling of mice and *P. berghei* parasites

*P. berghei* parasites were stored as frozen stocks at −80°C. Vial stocks were prepared by mixing 100 μL parasitized mouse blood with 100 μL of 30% glycerol in phosphate buffered saline. Mice were infected by intraperitoneal injection of 200 μL of thawed stocks or blood from animal donors. For parasite cloning it was also infected animals by intravenous injection. The parasitaemia was followed daily through Giemsa staining blood smears in glass slides. All *P. berghei* strains were maintained in 2–6 week-old female Balb/c mice. Animals were provided by the vivarium of the Institute of Biomedical Sciences and the vivarium of the School of Medicine of the University of São Paulo. Animals were housed in rooms with controlled temperature and humidity and a 12-hour light/dark cycle.

### 5.6 *P. falciparum in vitro* culture, parasite synchronization and knockout induction

In this study, we used the *P. falciparum* 3D7 strain Δ*Pf*PolK parasites. Both strains were cultured *in vitro* following the Trager and Jensen culture method employing RPMI-1640 medium completed with 0.5% Albumax I into 75 cm^2^ or 25 cm^2^ cell culture flasks at 37 °C (Trager & Jensen, 1976; Radfar et al, 2009; Crispim et al, 2022). The culture medium pH was adjusted to 7.4 and was introduced a gas mixture of 5% CO_2_, 5% O_2_ and 90% N_2_ purchased from Air Products Brasil LTDA^®^ (São Paulo, SP, Brazil). Parasites synchronization at ring stages was performed with 5% (w/v) D-sorbitol solution as described previously (Lambros & Vanderberg, 1979). Parasite development was monitored by Giemsa-stained smears microscopy. PCR for mycoplasma and optic microscopy were used to avoid culture contamination (Rowe et al, 1998).

### 5.7 Knockout induction of Δ*Pf*PolK *P*. *falciparum* parasites

The excision of the floxed *Pf*PolK-loxP in Δ*Pf*PolK parasites was carried out by adding 50 nM rapamycin or DMSO (used as a vehicle control) to synchronized ring-stage cultures. The cells were treated for 24 h, followed by a washout step. Subsequently, parasites were cultured again without rapamycin for an additional 24 or 48 h to begin IC_50_ assays (Crispim et al., 2024). For proteomic studies, the excision of the floxed *Pf*PolK gene was carried out three parasitic cycles before collecting samples.

### 5.8 Metabolic labelling of *P. falciparum* proteins

1 mL of synchronous cultures of *P. falciparum* at the ring stage was cultured in 12-well plates with either 0.75 μCi/ mL of each [^3^H] FOH or 40 μCi/ mL [^14^C] isoleucine. After 12–16 h, erythrocytes were centrifuged in 1.5 mL microtubes at 2000 × *g* for 10 min, and parasites at the trophozoite/schizont stages were obtained through saponin lysis (Christopher & Fulton, 1939). For this, cultures pellets were lysed with 1 mL 0.03 % saponin in PBS at 4 °C. Parasites were then centrifuged at 1,500 × g for 5 min at 4 °C and subsequently washed in PBS.

### 5.9 *Ex vivo* metabolic labelling of *P. berghei* proteins

*Ex vivo* culture of *P. berghei* parasites was performed based on the methods described by Janse et al., in 2006 and Lacroix et al., in 2011. For this, it was employed filter-sterilized RPMI1640 medium (with L-glutamine and 25 mM HEPES) supplemented with FBS 20% (vol/vol) and 50 μg / L neomycin-sulfate. For each animal at approximately 15% parasitaemia, 1 mL of infected blood was obtained by intracardiac puncture. Blood of each animal was divided in two aliquots which were immediately washed with 10 mL RPMI media and then centrifuged at 900 × *g*. Erythrocytes were suspended in 5 mL RPMI medium plus 0.75 μCi/ mL of [^3^H] FOH or 40 μCi/ mL [^14^C] isoleucine. The sample was introduced in 50 mL Falcon tubes filled with 5% CO_2_, 5% O_2_ and 90% N_2_ purchased from Air Products Brasil LTDA^®^ (São Paulo, SP, Brazil). Tubes and shaken at 36.5 °C for 16–18 h at a speed just enough to keep the cells in suspension. Finally, schizont stages were purified by saponin lysis (Christopher & Fulton, 1939). For this, cultures pellets were lysed with 30 mL 0.03 % saponin in PBS at 4 °C. Parasites were then centrifuged at 1,500 × g for 5 min at 4 °C and subsequently washed in PBS.

### 5.10 Analysis of radiolabelled proteins

The assessment of radiolabeled proteins was performed by 10% SDS-PAGE gels as described elsewhere (Verdaguer et al., 2022a). Briefly, radiolabelled parasites were sonicated thrice for 10 s at 50-W pulses in ice. Protein concentration was determined by BCA protein assay (Thermo Scientific) in 96-well flat bottom plates (Harow et al., 2006). The amounts of protein within controls and test samples was adjusted and solubilized in Laemmli buffer. Then, the protein extracts were boiled for 5 min at 95 °C and applied to each well for analysis. Gels were stained with Coomassie blue, treated with Amplify (Amersham), dried, and exposed to radiographic films with intensifying screen sets at −70°C. The contrast and brightness of autoradiography scans was adjusted for clarity.

### 5.11 Assessment of [^3^H] FOH incorporation into proteins

Assessment of [^3^H] FOH incorporation into parasite’s proteins was performed as described elsewhere (Verdaguer et al., 2022a). The parasites labeled with the radioactive marker were resuspended in 100 μL of a lysis solution composed of 2% w/v SDS, 60 mM DTT, and 40 mM Tris-Base at pH 9. Following cooling to room temperature, proteins were precipitated by adding 20% trichloroacetic acid (TCA) in acetone, chilled to 4°C. The mixture was kept on ice for 5 minutes before proteins were isolated by centrifugation at 12,000 × g for 10 minutes. The resulting pellet was rinsed three times with 80% acetone. The proteins were then dissolved by heating at 90°C for 30 minutes in an alkaline solution containing 0.5 M NaOH, 25 mM EDTA, and 0.1% w/v SDS in water. To complete the process, 1 mL of liquid scintillation fluid (PerkinElmer Life Sciences, MA, USA) was added. After vortexing, the samples’ radioactivity was determined using a Beckman LS 5000 TD β-counter (Beckman, CA, USA), with data analysis performed using GraphPad Prism software.

### 5.12 Assessment of [^3^H] GGOH incorporation into *P. falciparum* parasites.

Assessment of [^3^H] GGOH incorporation into *P. falciparum* parasites was performed as described elsewhere (Verdaguer et al., 2022a). For this, synchronous cultures were used at minimum 15% parasitaemia in the mature/schizont stage. Infected erythrocytes were transferred to 50 mL tubes and washed once with PBS. The supernatant was removed and the pellet containing only erythrocytes was distributed into 1.5 mL microtubes, 100 μL per tube. Then, tracer solution was added corresponding to 300 μM of the non-radioactive GGOH and 1 μCi/ mL [^3^H] GGOH. In some samples it was added different concentrations of geraniol and the total amount of the vehicle (ethanol) was adjusted between all samples. After 30 min incubation 1 mL of ice-cold PBS was added to stop the transport process. The samples were then washed 2 times in ice-cold PBS and suspended in 0.03% saponin, homogenized vigorously, and centrifuged. This extraction process was repeated three times to isolate parasites from erythrocytes. After the last centrifugation, the sample pellets were washed in PBS and suspended in Ultima Gold^®^ (Perkin Elmer^®^, Waltham, Massachusetts, United States) scintillation cocktail. The count of detected ionization events per minute (counts per minute, CPM) was monitored on a Beckman LS 5000 TD β-counter liquid scintillation spectrometer (Beckman^®^, CA, United States). The incorporation profiles were plotted and analysed with the help of GraphPad^®^ (GraphPad Software^®^, Inc., CA, United States) software. All centrifugation steps were 30 s at 10,000 × g at room temperature. In all experiments, a control was performed where the 0.3 mM non-radioactive GGOH solution was added, and immediately afterward the transport was stopped by the addition of ice-cold PBS (Time zero transport). The values in CPMs of this sample were discounted from the others. Three technical replicates were performed for each experiment. A near-linear incorporation of GGOH is observed during the initial 60 min of incubation, as previously reported (Verdaguer et al. 2022a). Leveraging this observation, we settled that the initial transport velocity (V_0_) in nmol.min^−1^.10^6^ schizonts, could be accurately estimated by measuring the incorporation of GGOH over a 30-minute period. This approach enabled the calculation of transport velocities under different conditions, providing insights into the inhibition of GGOH uptake.

### 5.13 Generation of Δ-*Pb*PolK *P. berghei* parasites

To generate the targeting sequence to knockout the PolK encoding gene in *P. berghei* (PBANKA_1220900, homologous to the experimentally validated *P. falciparum* sequence PF3D7_0710300) we employed the same procedure described elsewhere (Lacroix et al., 2011; Bargieri et al., 2016). First, two homology regions (HR) fused to restriction sites were amplified from *P. berghei* gDNA and further cloned in a DcOmCherry plasmid to promote homologous recombination: the PolK 5′UTR (962 bp; hereafter referred as HR1) and 3′UTR (848 bp; hereafter referred as HR2) flanking a cassette containing PbEf1a promoter for hDHFR sequence followed by 3’PbDHFR-TS; PbHSP70 promoter preceding mCherry sequence and 3’PbHSP70. *P. berghei* genetic transfection, pyrimethamine selection and cloning was performed exactly as described by Lacroix et al., 2011. The gDNA amplification of the two homology regions was performed by PCR using primer pairs HR1–5’_P.Foward-KpnI (5’ CCCGGTACCACATTCTATTTCCGAATCGTT 3’) plus HR1–5’_P.Reverse-XhoI (5’ GGGCTCGAGCAAACAATATTGTGTAGAAAC 3’) or HR2–5’_P.Foward-BglII (5’ CCCAGATCTCATAAATAACACATTCCCTATT 3’) plus HR2–5’_P.Reverse-EcoRI (5’ GGGGAATTCAACAAGAAGTTCTTATAAATCC 3’). The plasmid DcOmCherry+HR1+HR2 was sequenced by routinely procedures. The detection of native *Pb*PolK gene was performed by PCR using the primer pair Int-WT-forward (5’ GATCGGCAATCTGAGCATTC 3’) and Int-WT-reverse (5’ AGGAATTTGGGAACACACATCT 3’) which amplify a 1438 bp sequence. The detection of recombinant locus containing mCherry sequence was performed by PCR using the primer pair Int-Δ*Pb*PolK-forward (5’ GCTTGTTTTGTGAATTCGTG 3’) and Int-Δ*Pb*PolK-reverse (5’ TGGTGCTTTGAGGGGTGAGC 3’) which amplify a 1233 bp sequence. *P. berghei* genetic manipulation and all molecular biology procedures necessary for this purpose were performed as described by Lacroix et al., (Lacroix et al., 2011).

### 5.14 Fluorescence microscopy

Parasite fluorescence microscopy and flow cytometry was performed using 5 μL of blood samples, as described elsewhere (Bargieri et al., 2016; Marin-Mogollon et al., 2019). Samples were suspended in 1 mL PBS and stained with the DNA-specific dye Hoechst-33342 (Sigma Aldrich, St Louis, MO, USA) by adding 20 μL of a 500 μM stock-solution (final concentration 10 μM) for 20 min at 37°C. Subsequently, 5 μL were placed on a microscopic slide under a cover slip and images of fluorescent cells were acquired with a digital DFC 365 FX camera coupled to a DMI6000B/AF6000 microscope (Leica) at x1000 magnification.

### 5.15 *In vivo* treatment and statistical analyses

Mice infected with either wild-type parasites or transgenic parasites were randomly assigned to one of the experimental groups. Three mice were used for each treatment group, and two mice were used for the control groups which received drugs or the corresponding drug vehicles, as indicated. All experiments were performed three times. Mice were infected by intraperitoneal injection with 1 × 10^7^ infected erythrocytes from a donor mouse. In this study it were employed several drugs: Fosmidomycin was dissolved in phosphate-buffered saline and administered intraperitoneally at a dose of 50 mg/kg of body weight every 24 hours, or at 25 mg/kg every 12 hours (Walker, 2018). In both cases, the treatment was administered on days 4–5 post-infection. Artesunate was dissolved in phosphate-buffered saline and given orally as a single dose of 2.4 mg/kg of body weight on day 4 post-infection. Geraniol, prepared as an emulsion in glycerol, was administered orally at 120 mg/kg of body weight every 12 hours on days 4–5 post-infection. The doses of artesunate and fosmidomycin described here correspond to sub-therapeutic treatment, as previously determined by other authors (Wiesner et al., 2002; Osirime et al., 2020). The dose of geraniol corresponds to non-toxic and pharmaceutically acceptable treatments, as previously determined by other authors (Pavan et al., 2018). Parasitaemia was monitored through tail blood smears every two days, and mice were euthanized when it exceeded 40%. To compare the means of variables, the unpaired student’s t-test was employed. In addition, survival curves were plotted using the Kaplan-Meier method, and statistical differences were evaluated using the log-rank test. The analyses were performed using the GraphPad PRISM^®^ 5.3 program.

### 5.16 Monitoring *P. falciparum* growth

The dose–response curve and the concentration of drug/metabolite required to cause a 50% reduction in parasite growth (IC_50_ value) was estimated by DNA staining as described elsewhere (Smilkstein et al, 2004). Briefly, assays started at ring stage at 1% parasitaemia and had a duration 72h. Serial dilutions of each drug were prepared in 96-well microplates in RPMI complete medium sometimes supplemented with terpene compounds as indicated. Solvent controls and untreated controls were always included. After the incubation time, parasite growth was monitored by incubating 100 μM of the culture with 100 μM of an SYBR Green I^®^ DNA staining solution at 1:20,000 final dilution in lysis buffer (20 mM Tris, pH 7.5; 5 mM EDTA; 0.008% saponin (v/v); 0.08% Triton X-100 (v/v), the fluorescence was measured in a POLARstar Omega fluorometer^®^ (BMG Labtech^®^, Ortenberg, Germany) with the excitation and emission bands centered at wavelengths of 485 and 520 nm respectively. Finally, the values obtained were subtracted from those values obtained for uninfected erythrocytes suspended at the same hematocrit. Solvent controls and untreated controls were always included and results were analyzed by GraphPad Prism^®^ software. Inhibition of parasite growth was analyzed with respect to the logarithm of the concentration using nonlinear regression (dose-response slope/variable sigmoid equation). All experiments were performed at least three times with four technical triplicates for each one.

### Parte inferior do formulário

### 5.17 Recombinant expression in yeasts

Recombinant expression of *Pf*PolK (PfNF54_070015200) was performed in *Saccharomyces cerevisiae* W303–1A strain -an organism without POLK enzymes- as described elsewhere (Crispim et al., 2024). Briefly, the gene *Pf*PolK was purchased Genscript already optimized for its expression in yeast. *Pf*PolK was cloned in a p416-GPD vector (p416-GPD-*Pf*PolK) and transformed into yeasts by the lithium acetate methodology (Gietz & Woods, 2002). Cells were routinely cultivated in liquid 2% dextrose Synthetic Defined medium without addition of uracil (SD-Ura) (Bergman, 2001).

### 5.18 Farnesol kinase activity assays

The recombinant membrane-bound PolK was assayed in crude extracts of yeasts transformed with p416-*Pf*PolK, as described elsewhere (Crispim et al., 2024). Briefly, yeasts were cultured until early stationary phase in SD-Ura plus dextrose medium. Then, cells were disrupted by glass beads (0.5 mm of diameter) (Avramia & Amariei, 2022). Unbroken cells were discarded by centrifugation at 900 × *g* for 1 min and protein was adjusted to 50 mg/ mL with 100 mM Tris/HCl pH 7,4. The reaction was performed in 1.5 mL microtubes by incubating approximately 40 mg of yeast protein with 4 mM MgCl_2_, 800 μM CTP, 10 mM sodium orthovanadate, 0.05% 3-[(3-Cholamidopropyl) dimethylammonio]-1-propanesulfonate (CHAPS) and 2 μCi [^3^H] FOH. [^3^H] FOH was vacuum-dried as it is commercially distributed in ethanol. The volume was adjusted to 100 μL with 100 mM Tris/HCl pH 7,4 and the reaction was initialized by adding the yeast extract. In some cases, CTP addition was omitted. In other cases, it was added drugs to the reaction, employing DMSO as vehicle (0.5% final concentration, DMSO was also tested to not affect the enzymatic activity). After 30 min of incubation at 37 °C the reaction was stopped by adding 500 μL of n-butanol saturated in water. The mixture was vortexed, centrifuged at 12.000 × *g* for 10 min and the organic phase was dried under vacuum. The residue was suspended in 10 μL of n-butanol saturated in water and chromatographed on silica 60 plates (20×20 cm, Merck). Plates were developed for 7–10 cm with isopropyl alcohol/ammonia (32%)/water (6:3:1 by volume). Authentic standards of FOH / GGOH and its respective phosphates and pyrophosphates were run in other plates and visualized with iodine vapour in order to identify the Retention factor (R*f*) of the reaction products and substrates. Finally, the plates were treated with EN3HANCE (Perkin Elmer) and exposed to autoradiography for several days at −70°C. The contrast and brightness of autoradiography scans were adjusted for clarity. To perform a quantitative pixel analysis of bands from FP autoradiographic images, the individual images were imported into the ImageJ software (National Institutes of Health, USA) and converted into RGB format for analysis. Initially, it was selected the first lane using the “Rectangle” tool. This selection was then defined as the initial lane for analysis via the menu pathway: “Analyze” > “Gels” > “Select First Lane.” Subsequent lanes were selected similarly, with each being designated through the pathway “Analyze” > “Gels” > “Select Next Lane,” continuing this process until the final lane was reached. For the pixel analysis, we selected the “Analyze” > “Gels” > “Plot Lanes” option, which facilitated the visualization of pixel distribution across the image via a graph displaying distinct peaks. Detailed quantification of pixel density was conducted by estimating each peak area using the “Wand (tracing)” tool. To calculate the percentage of produced FP, we determined the ratio of pixels corresponding to FP in enzymatic assays relative to the positive control—the latter being deemed representative of 100% FP production. From three separate experiments, it was computed the mean and standard deviation (SD).

### 5.19 Extraction of lipids of blood plasma

Lipids from 0,5 mL of mouse plasma were extracted following a protocol based on the studies cited herein (Crispim et al., 2024). Samples were extracted with 9 mL of Chloroform/Methanol (2:1 by volume), mixed, and centrifuged at 1,000 × *g* for 2 min. The lower lipid-containing organic phase was dried under nitrogen stream. The residue was dissolved in 0,5 mL of Ethanol and hydrolyzed with 0,5 mL of 5M KOH at 56°C for 1 hour. After cooling and neutralizing with 0,5 mL of 5M HCl, the solution was partitioned with 2.4 mL of n-Hexane, 0.6 mL of Water, and 0.6 mL of Ethanol. The upper organic phase was evaporated to dryness and suspended in 100 μL of Ethyl acetate for further mass spectrometry analysis.

### 5.20 Mass spectrometry analysis

Mass spectrometry analysis of mouse plasma lipids was performed based on the studies cited elsewhere (Kai et al., 2018; Crispim et al., 2024). Briefly, FOH and GGOH standards at 1 μM were prepared in Ethyl acetate. The GC-MS triple quadrupole system consisted of a Nexis GC-2030 gas chromatograph coupled with tandem TQ8050 NX mass spectrometer and AOC-5000 auto injector (Shimadzu, Kyoto, Japan). The system was controlled by GC-MS Real Time Analysis software, version 4.53 and the spectra were manipulated using GC-MS Postrun Analysis software, version 4.53. The GC analysis were performed on a Shimadzu column SH-200MS (30m, 0.25 mm I.D., 0.25 μm film thickness), the chromatographic conditions were: inlet temperature 230°C, pressure 59.8 kPa, total flow 21.6 mL/min, column flow 1.03 mL/min, using helium (purity = 99.999%) as the carrier gas. The temperature was initially held at 60°C for 1 min, them increased to 160°C at a rate of 25°C/min, finally by a 12°C/min ramp to 300°C and held by 2 min. Triple-quadrupole MS mass spectrometer was operated at interface and ion source temperatures of 280°C, solvent delay 3 min and using collision gas argon (minimum purity 99.9999%). The mass spectrometer was working in EI mode of 70 eV. The optimal quantitation and confirmation transitions from parent ions to daughter ions and collision energy for MRM of each compound were achieved with Auto-MRM study tests performed by the software (MRM transition of GGOH and FOH in Table 1).

### 5.21 Molecular modelling

*Pf*PolK model was retrieved from our previous publication (Crispim et al., 2024), as well as data on the substrates. All potential inhibitor for docking were drawn using Maestro and prepared using LigPrep to generate the three-dimensional conformation, adjust the protonation state to physiological pH (7.4), and calculate the partial atomic charges, with the force field OPLS4, for racemates both (*R*) and (*S*) were considered separately. Docking studies with the prepared ligands were performed using Glide (Glide V7.7), with the flexible modality of Induced-fit docking (Sherman et al, 2006; Friesner et al, 2006) with extra precision (XP), followed by a side-chain minimization step using Prime. Ligands were docked within a grid around 13 Å from the centroid of the GGOH pocket, previously proposed by us (Crispim et al., 2024), generating ten poses per ligand. Docking poses were visually inspected, independently from the docking score, and those with the highest number of consistent interactions were selected for simulation.

### 5.22 Molecular dynamics simulations

*Pf*PolK model with the different inhibitors was simulated to clarify which residues contributed to the stability within the binding site. Molecular Dynamics (MD) simulations were carried out using the Desmond engine (Bowers et al, 2006) with the OPLS4 force-field (Lu et al, 2021). The simulated system encompassed the protein-ligand/cofactor complex, a predefined water model (TIP3P) (Jorgensen et al, 1983) as a solvent, counterions (Na^+^ or Cl^−^ adjusted to neutralize the overall system charge) and a POPC membrane based in the transmembrane motifs determined in the model. The system was treated in an orthorhombic box with periodic boundary conditions specifying the shape and the size of the box as 10×10×10 Å distance from the box edges to any atom of the protein. Short-range coulombic interactions were performed using time steps of 1 fs and a cut-off value of 9.0 Å, whereas long-range coulombic interactions were handled using the Smooth Particle Mesh Ewald (PME) method (Darden et al, 1993). PolK + CTP + potential inhibitors systems were then subjected to simulations of 100 ns for equilibration purposes, from which the last frame was used to generate new replicas. The equilibrated system underwent at least 500 ns production simulation, in five replicas (total of 2.5 μs per substrate), followed by analysis to characterize the protein-ligand interaction. The results of the simulations, in the form of trajectory and interaction data, are available on the Zenodo repository (code: 10.5281/zenodo.10925367). MD trajectories were visualized, and figures were produced using PyMOL v.2.5.2 (Schrödinger LCC, New York, NY, USA). Protein-ligand interactions and distances were determined using the Simulation Event Analysis pipeline implemented using the software Maestro 2024v.1 (Schrödinger LCC). The compounds’ binding energy was calculated using the Born and surface area continuum solvation (MM/GBSA) model, using Prime (Jacobson et al, 2004) and the implemented thermal MM/GBSA script. For the calculations, each 50^th^ frame of MD was used. Finally, root mean square deviation (RMSD) values of the ligand’s heavy atoms were used to cluster the trajectory and identify relevant binding modes.

### 5.23 Proteomics

Parasites were purified of lipids and other contaminants by SDS-PAGE. For this purpose, it was used 50 μg of parasite protein solubilized in Laemmli Buffer per sample, prepared as described before. Three replicates for each parasite strain extracts were loaded on a 10% SDS-PAGE and run for 1–2 cm. The gel bands containing all proteins were then excised and proteins were in-gel digested as previously described (Shevchenko et al. 2006). Briefly, gel bands were distained with 40% acetonitrile in 50 mM ammonium bicarbonate (AMBIC), were reduced with 10 mM 1,4-Dithiothreitol (DTT) in 50 mM AMBIC for for 45 min at 56°C, alkylated with 55 mM iodoacetamide in 50 mM AMBIC 30 min at room temperature, protected from light Protein was digested into peptides by trypsin (Promega) at 1:50 (w/w) at 37°C for 16 h. The reaction was stopped with trifluoroacetic acid (TFA) at a 1% final concentration. The peptides were extracted from gel using 40% acetonitrile and 0.1% TFA and were purified on a house made C18 stagetips (Thermo Scientific) (Gobom et al., 1999; Larsen et al., 2002). The peptides were dried under a vacuum centrifuge, and stored at −20 °C for further analysis. The samples analysis were performed at Biomass—Mass Spectrometry and Proteome Discovery Facility at *Centro de Facilidades à Pesquisa* (CEFAP-USP) of the Institute of Biomedical Sciences of the University of São Paulo, using an EASY-nano LC system (Thermo Scientific) coupled to a LTQ-Orbitrap Velos mass spectrometer (Thermo Scientific). Peptides were loaded into a C18 PicoFrit column (C18 PepMap, 75 μm id × 10 cm, 3.5 μm particle size, 100 Å pore size; New Objective, Ringoes, NJ, USA) and eluted at a flow rate of 300 nL/min using a linear gradient of 2–30% phase B (0.1% formic acid, 95% acetonitrile) for 105 min. Mass spectra were acquired in positive ionization mode using a data dependent acquisition method where the top 20 most intense precursor ions at MS1 spectra with charge-state ≥2, acquired at a 60,000 FWHM resolution, 400–1500 m/z mass range and a minimum threshold of 1×10^6^ were selected to fragmentation using CID at 35 normalized collision energy, 10 ms activation time and 30 s of dynamic exclusion. The raw data were accessed in Xcalibur v.2.1 (Thermo Scientific) and data processing was performed using MaxQuant version 1.5.3.8 for protein identification and quantification, using Andromeda search engine against *P. falciparum* 3D7 strain database downloaded from PlasmoDB (https://plasmodb.org/plasmo/app, last accessed in April 2024) combined with human protein sequences. The analysis was performed with the following parameters: enzyme specificity was defined as full trypsin, allowing up to two missed cleavages. Carbamidomethylation of cysteine (57.021 Da) was set as a fixed modification, oxidation of methionine (15.994 Da) and N-terminal protein acetylation (42.010 Da) were included as variable modifications. The precursor ion tolerance level was 20 ppm for the first search and 4.5 ppm for the main search. A false discovery rate (FDR) of 1% was applied at peptide and protein level. The “match between run” option was enabled. Protein quantification was performed using label-free quantification (LFQ) by integrating the extracted ion chromatogram (XIC) area. The protein expression data were processed using Perseus 2.0.7.0 computational platform (Tyanova et al., 2016). LFQ Intensities data were log2 transformed, protein reverse, contaminants were removed and the proteins data was filtered by at least two valid values in at least one experimental group. Imputation was performed where missing values were replaced by random values drawn from a normal distribution.

Exclusive proteins were defined as those identified in all three replicates of one condition and not detected in any replicate of the other (with or without *Pf*PolK preservation in parasites). The analysis of protein abundance changes between the conditions was performed using two different statistical methodologies. The more restrictive method involved an unpaired Student’s t-test comparation between the conditions, using Benjamini-Hochberg FDR correction. Proteins with an FDR < 0.05 were considered regulated proteins. Alternatively, the less restrictive method involved an only unpaired Student t-test, where proteins with a p-value < 0.05 were considered differentially abundant proteins. Hierarchical clustering analysis was performed after Z-score normalization. Gene Ontology Biological Process and Cell Component analysis were performed using the tools available on PlasmoDB (https://plasmodb.org/plasmo/app, last accessed in April 2024). Specifically, the PlasmoDB accession numbers were employed to find the GO terms of regulated/exclusive proteins. For this, it was performed a Gene Ontology Enrichment of biological processes, with evidence computed or curated, limiting to GO Slim terms (p-value cutoff = 1). Finally, MS data is available for public access in the ProteomeXchange Consortium *via* the PRIDE repository with the dataset identifier num. PXD059094.

## Figures and Tables

**Table 1.** MRM transition of FOH and GGOH.

Compound	RT (min)	MRM transition 1	Collision Energy	MRM transition 2	Collision Energy	MRM transition 3	Collision Energy
		Quantifier	eV	Qualifier	eV	Qualifier	eV

FOH	7.88	93.00>77.00	12	81.00>79.10	9	93.00>91.10	6

GGOH	10.97	93.00>77.00	12	81.00>79.10	9	93.00>91.10	6

The table displays the MRM transition of the FOH and GGOH for GC-MS/MS analysis. RT: Retention time, MRM: Multiple reaction monitoring.

## Abbreviations

CHAPS: 3-[(3-Cholamidopropyl)dimethylammonio]-1-propanesulfonate
CTP: Cytidine 5′-triphosphate
DMAPP: Dimethylallyl pyrophosphate
FOH: Farnesol
FPP: Farnesyl pyrophosphate
FP: Farnesyl monophosphate
GGPP: Geranylgeranyl pyrophosphate
GGP: Geranylgeranyl monophosphate
GGOH: Geranylgeraniol
IPP: Isopentenyl pyrophosphate
POLK: Prenol kinase
PPP: Prenyl-PP phosphatase
PT: Prenol transporter
SDS-PAGE: Sodium dodecyl sulfate–polyacrylamide gel electrophoresis
MRM: Multiple-reaction monitoring
GC/MS: Gas chromatography–mass spectrometry
DTT: 1,4-Dithiothreitol
